# No Difference in Return-to-Sport Rate or Activity Level in People with Anterior Cruciate Ligament (ACL) Injury Managed with ACL Reconstruction or Rehabilitation Alone: A Systematic Review and Meta-Analysis

**DOI:** 10.1007/s40279-025-02268-5

**Published:** 2025-07-02

**Authors:** Stephanie R. Filbay, Garrett Bullock, Scott Russell, Frances Brown, Wilson Hui, Thorlene Egerton

**Affiliations:** 1 Centre for Health Exercise and Sports Medicine, Department of Physiotherapy, The University of Melbourne, Level 7, Alan Gilbert Building, Melbourne, VIC 3010 Australia; 2 Department of Orthopaedic Surgery and Rehabilitation, Wake Forest University School of Medicine, Winston-Salem, NC USA; 3 Department of Physiotherapy, The University of Melbourne, Melbourne, VIC Australia

## Abstract

**Background:**

A common belief amongst patients and clinicians is that anterior cruciate ligament reconstruction is required to return to sport. It is not clear if this belief is supported by the best available research.

**Objective:**

We aimed to compare return-to-sport and activity levels following anterior cruciate ligament rupture managed with anterior cruciate ligament reconstruction versus rehabilitation alone.

**Methods:**

We performed a systematic review and meta-analysis. A comprehensive search was conducted across seven electronic databases for empirical studies published to July 2023. Articles were included if they assessed return-to-sport and/or activity levels in two groups where one underwent an anterior cruciate ligament reconstruction and the other had exercise-based rehabilitation that was standardised and/or supervised by a healthcare professional. In addition to narrative syntheses, random-effect meta-analyses were conducted for return-to-sport and activity participation (Tegner Activity Scale). The protocol was pre-registered (PROSPERO CRD42022313507).

**Results:**

Eighteen articles reporting on 15 studies (two randomised controlled trials) met inclusion criteria. Ten studies had a high risk of confounding bias that was likely to favour anterior cruciate ligament reconstruction including biases in group allocation and differences in activity and return-to-sport advice between groups. The findings suggest that anterior cruciate ligament reconstruction was not associated with higher return-to-sport rates (odds ratio 1.5, 95% confidence interval 0.76–2.97) compared to rehabilitation alone. A small difference favouring anterior cruciate ligament reconstruction was observed for Tegner Activity Scale scores (mean difference 0.7, 95% confidence interval 0.16–1.24) that did not exceed the minimal detectable change and no difference was observed after excluding studies with a high risk of confounding bias. Insufficient data were available for time to return to sport and physical activity levels. The evidence is of low or very low certainty because of the heterogeneity of results and the high risk of bias in the included studies.

**Conclusions:**

There was no difference in return-to-sport rates or activity levels when comparing anterior cruciate ligament reconstruction with rehabilitation alone for the management of anterior cruciate ligament injury.

**Supplementary Information:**

The online version contains supplementary material available at 10.1007/s40279-025-02268-5.

## Key Points

**Table:** 

Return-to-sport rates were similar irrespective of management of anterior cruciate ligament rupture with anterior cruciate ligament reconstruction or rehabilitation alone (very low certainty evidence).
No meaningful difference in activity levels was observed between treatment groups, assessed with the Tegner Activity Scale, which considers both the level of competition and the demands of the sport (very low certainty evidence).
Similar return-to-sport and activity levels between treatment groups were found despite most studies having a high risk of confounding bias favouring the anterior cruciate ligament reconstruction group (i.e. they recommended non-surgical management if patients were less active pre-injury or did not plan on returning to sport, and/or only advised people in the non-surgical group that they should not return to sport).
Only one study compared time to return to sport or physical activity participation between groups. No differences were observed between groups, but more research is needed.

## Introduction

As many as 91% of people expect to return to sport after undergoing anterior cruciate ligament reconstruction (ACLR) [1]. However, the latest meta-analysis found that 65% of people returned to pre-injury sport and only 55% of people returned to competitive sport after ACLR [2]. Despite this, a recent consensus statement recommends ACLR as the preferred treatment to maintain sports participation after anterior cruciate ligament (ACL) injury [3]. This recommendation was based on expert opinion, rather than on evidence. People who do not return to sport after ACLR report poorer long-term quality of life [4] and some adopt an inactive lifestyle [5]. Adopting an inactive lifestyle has health and well-being implications, and may exacerbate an already elevated risk of knee osteoarthritis, particularly when associated with an increase in body mass [6] and chronic systemic inflammation [7].

An alternative to ACLR is management with exercise-based rehabilitation. Recommended exercise-based rehabilitation comprises a phase-based approach where progression is determined by the attainment of milestones rather than time post-injury [8]. This should include exercises that target strength, neuromuscular control, proprioception/balance, agility, power, perturbations, functional tasks and sport-specific skills, and includes the management of any knee concerns (e.g. range-of-motion deficits, pain, swelling, reinjury fears) [8]. Knee pain, other knee symptoms, sports and recreational function, and quality-of-life outcomes from exercise-based rehabilitation are comparable to ACLR at 2 and 5 years after an acute ACL injury [9–11]. In Australia, USA, Canada and the UK, most people with ACL injury undergo ACLR despite recommendations to trial exercise-based rehabilitation as the first-line treatment in most cases [12, 13]. Returning to sport is often the main goal for people after ACL injury, and advice from healthcare professionals that ACLR is needed in order to return to sport may contribute to the high preference for ACL surgery. A synthesis of the evidence comparing return to sport and activity levels between ACL treatment strategies, considering research bias and quality, is clearly needed to inform clinical decision making and the advice provided to patients. Most systematic reviews that have evaluated return-to-sport and/or activity levels after ACL injury have only reported outcomes for people managed with ACLR [2, 14, 15], or classified non-surgical treatment as the absence of ACLR rather than treatment with rehabilitation alone [16, 17]. Other existing reviews are limited by including only randomised controlled trials (RCTs) despite a lack of RCT evidence and no existing RCTs with return to sport or activity level as a primary outcome [12]. The aim of this systematic review and meta-analysis is to answer the following question: Is there a difference in return-to-sport and activity levels in individuals with an ACL rupture that were managed with ACLR versus exercise-based rehabilitation alone?

## Methods

The study is reported according to the Preferred Reporting Items for Systematic Reviews (PRISMA) guidelines [18]. The study was prospectively registered with PROSPERO (CRD42022313507). Review management utilised Covidence (Covidence.org).

### Literature Search

Seven databases (MEDLINE [Ovid], Embase [Ovid], CINAHL, PEDro, SPORTDiscus, Cochrane Central Register of Controlled Trials and Scopus) were searched from inception to 20 July, 2023. The search strategy (Appendix 1 of the Electronic Supplementary Material [ESM]) consisted of keywords and MeSH (or equivalent) terms in three categories: (1) ACL/rupture/reconstruction; (2) rehabilitation; and (3) outcomes of interest to this review. The search strategy was peer reviewed using the Peer Review Electronic Search Strategy (PRESS) checklist [19] by a University of Melbourne librarian. Reference lists of included articles and systematic reviews on related topics, and clinical trial registers (ClinicalTrials.gov and World Health Organization International Clinical Trials Registry Platform) were searched for additional articles or leads.

### Study Selection

Randomised controlled trials and observational studies (such as cohort studies with two groups and cross-sectional studies with two groups) comparing ACLR with exercise-based rehabilitation alone, in participants with primary or secondary rupture of the ACL (with or without associated meniscal, other ligamentous and/or osteochondral injury), were eligible for inclusion. Case reports, case studies and single-group pre-post study designs were excluded, as were studies not published in peer-reviewed journals and those not written in English.

Studies where an autograft was used for ACLR were eligible for inclusion. However, studies where < 30% of the surgical group underwent primary ACL repair or ACLR with a synthetic graft (allograft), or where < 20% of participants had a bilateral injury or partial tear, were also eligible. To be included, comparison groups needed to have undergone non-surgical management of an ACL rupture comprising exercise-based rehabilitation that was standardised and/or supervised by a healthcare professional. Differences between groups in the quality or quantity of rehabilitation protocols, and/or in return-to-sport instructions, were not a reason for study exclusion; however, interpretation of findings from these studies was mindful of the potential for, and the likely direction of, bias in their findings.

To be included, articles must have reported return-to-sport outcomes (i.e. rates or time to return to sport), and/or activity levels (i.e. activity participation scales such as the Tegner Activity Scale, Marx scale or UCLA activity scale) or physical activity levels such as self-reported (e.g. International Physical Activity Questionnaire) or objective measures (e.g. accelerometry, step counts). As there is no agreed-upon method or gold-standard instrument for assessing return to sport after ACL injury, all methods of assessment were considered eligible for this review. At least two reviewers independently assessed each title/abstract and then the full texts of remaining articles against eligibility criteria (GB, SR, FB, WH, TE), with a third reviewer involved to resolve conflicts as required (SF).

A refinement to our study inclusion/exclusion criteria was made after our protocol was registered such that studies with data from early ACLR participants and delayed ACLR participants pooled together could be included. In contrast, studies in which data from delayed ACLR participants and data from rehabilitation-alone participants were pooled and not provided separately for each group were excluded. The rationale for this was: (a) several studies that otherwise met our inclusion criteria included participants who had their ACLR surgery longer or even much longer than 6 months after injury in their ACLR group and this spread of timing of surgery appeared similar to the delayed ACLR surgery groups in other studies and (b) pooling ACLR and delayed ACLR (but not delayed ACLR and rehabilitation alone) was consistent with our research question of comparing return-to-sport and activity levels after surgery versus no surgery.

### Risk of Bias Assessment

Risk of bias in included studies was assessed at the outcome level using the Cochrane Risk of Bias Tool Version 2 (ROB2) [20] for randomised controlled trials, or the Risk of Bias Assessment tool for Non-randomized Studies of Interventions (ROBINS-I) [21]. At least two reviewers independently assessed the risk of bias. Discrepancies were resolved through discussion or a third reviewer. The risk of bias identified in each domain was considered in relation to the likely direction of effect of each source of bias in individual studies when interpreting findings, for example, if groups were different at baseline because of the study methods or if treatment advice that could directly affect outcomes was different between groups. All outcomes of interest were participant reported, and it is not possible to blind participants to interventions in studies comparing surgery to no surgery. As awareness of the intervention received also reflects real life, we took a pragmatic approach and did not downgrade studies because of unblinding of assessors.

### Data Extraction

Data were extracted by one of the team of reviewers (SF, GB, SR, FB, WH or TE) into pre-defined spreadsheets, and all data independently checked against the original article by at least one other reviewer. Extracted data included article details (author, year), study characteristics (study design, number recruited into each group, number included in analysis), participants details (age, sex/gender, pre-injury sports participation, other study inclusion/exclusion criteria), type and dosage of interventions (surgical and rehabilitation management, other instructions given to participants), outcomes measured and timepoints (time from injury to surgery, time from injury/surgery to assessment of outcome) and results data by group. Attempts were made to contact article authors for missing data.

### Data Synthesis

Meta-analyses were performed for return-to-sport and activity-level outcome measures using random-effect models with inverse variance weighting and 95% confidence intervals (95% CIs), provided enough data were available from at least two studies. Random-effects models were chosen as it was assumed that effects could vary between studies because of study heterogeneity (e.g. differences in study eligibility criteria and interventions) [22]. Pooled odds ratio (95% CI) was estimated based on the proportion of participants who returned to sport, and pooled mean difference (95% CIs) was estimated for activity levels assessed with the Tegner Activity Scale score. Heterogeneity was assessed through the overall Tau score, *I*^2^ (heterogeneous if ≥ 50%). All analyses were performed in RevMan Web [23]. For studies eligible for a meta-analysis that only reported the median (interquartile range) of Tegner Activity Scale scores, the standard deviation (SD) was estimated using the sample size, median, range and/or interquartile range, as appropriate [24, 25]. Two studies only reported mean (range), but also reported the proportion of scores in each Tegner category [26, 27]. For these studies, median and interquartile range were calculated based on the spread of data, and the above method was then used to estimate SD for use in the meta-analysis. Data that could not be pooled in the meta-analysis are described narratively according to recommendations from SWIM guidelines for reporting synthesis without a meta-analysis [28].

Several sensitivity and sub-group meta-analyses were planned a priori if data allowed. These included: (1) a sub-group analysis comparing outcomes for participants who crossed over to delayed ACLR after initial rehabilitation versus those who remained managed with rehabilitation alone; (2) a sub-group analysis based on time since ACL injury (e.g. ≤ 5 years, 5–10 years and > 10 years); and (3) a sensitivity analysis excluding studies with a high risk of bias. The first sub-group analysis was not conducted because it was unclear which studies separately reported crossed-over participants versus included them in the ACLR group data, making interpretation of findings impossible. In addition, rather than conducting the pre-planned sensitivity analysis, a sub-group analysis was added where studies were grouped based on the risk of confounding bias. We considered this analysis of more value than a sensitivity analysis because of its importance for exploring the effect of confounding biases. Studies were considered to have a high risk of confounding bias if they (a) recommended people with a low pre-injury activity level to choose non-surgical management, (b) allocated people to non-surgical management if they expressed they did not aim to return to pre-injury sport and/or (c) only advised people in the non-surgical group that they should limit activity or not return to sport. All three confounding biases were assumed to disadvantage the rehabilitation-alone group in comparisons of activity participation and return-to-sport outcomes, and these studies were grouped together as one sub-group, with the remaining studies without these characteristics in the low risk of confounding bias sub-group.

### Confidence in Review Findings

The level of certainty in the review findings was determined based on the GRADE (Grading of Recommendations, Assessment, Development and Evaluations) criteria [29]. Level of certainty could be downgraded because of the risk of bias, inconsistency of findings, indirectness of source studies and/or imprecision of findings; or upgraded owing to a large effect, and/or if confounding variables or known study biases were likely to reduce a demonstrated effect [29].

## Results

### Study Characteristics

**Fig. 1:**
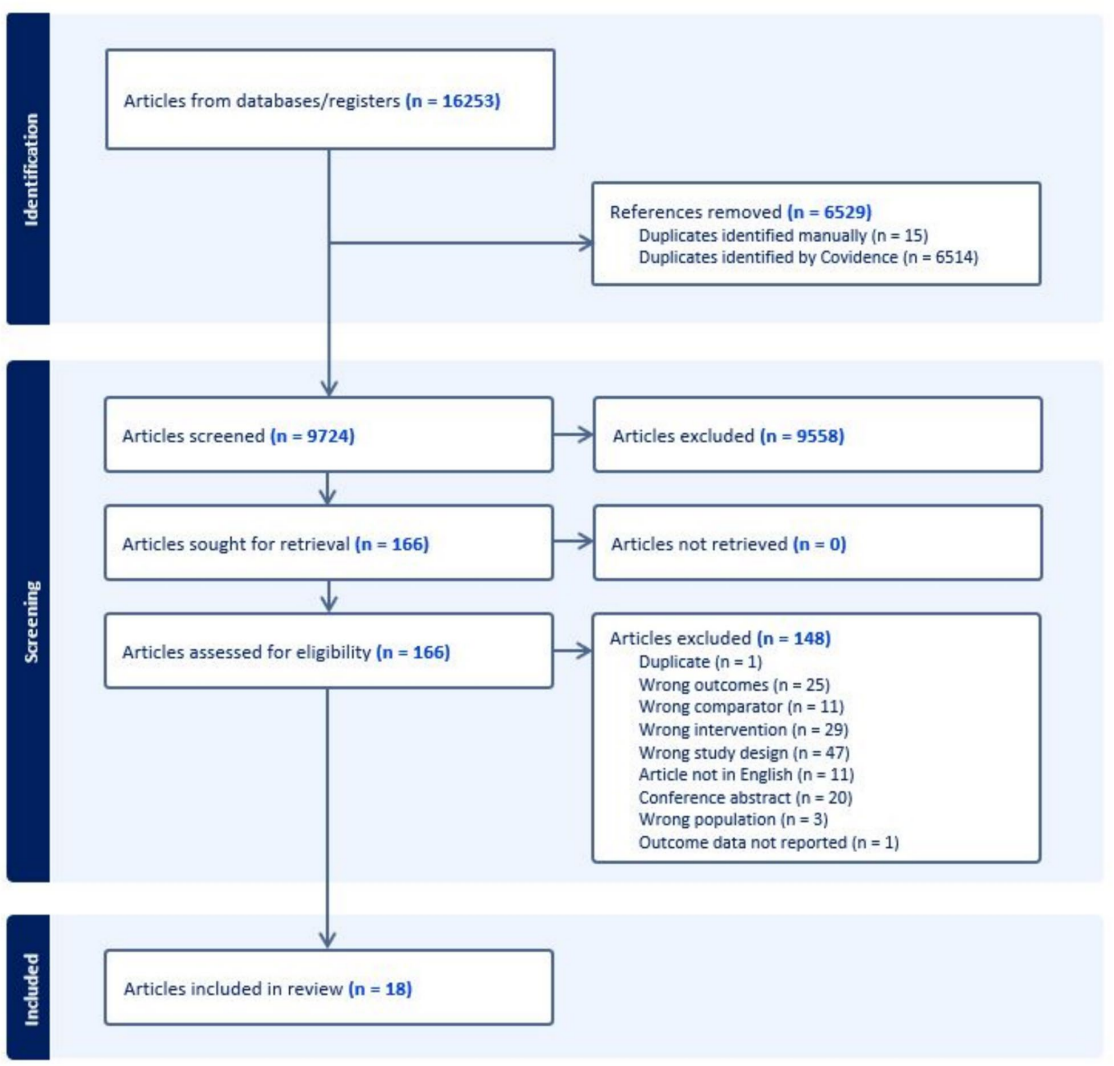
Preferred Reporting Items for Systematic Reviews (PRISMA) flow chart outlining article selection process and reasons for article exclusion

The study selection process and reasons for article exclusion are shown in Fig. 1 and study details and participant characteristics (including study design, sample size, age of participants, follow-up, details on treatment allocation/selection, return to sport recommendations, rehabilitation description, findings) are presented in Appendix 2 of the ESM. Eighteen articles, from 15 studies (including a total of 1637 participants, proportion of female participants ranging from 0% [30, 31] to 56% [32]), were eligible and included in this review. All studies included participants who had primary ACLR. Two articles included the same participants and data [33, 34], so the article [33] reporting fewer data was excluded from all analyses/syntheses. Two articles [10, 35] reported the long-term follow-up of studies reported in earlier articles [9, 36]. Data from the studies with follow-up assessments closest to 5 years [10, 36] were used in the primary analyses, as 5 years is likely to not be impacted by recency of surgery and was of the greatest clinical interest. Data from all articles were included in the sub-group analyses of different follow-up durations. Only three studies (four papers [9, 10, 37, 38]) reported outcomes separately for early ACLR, delayed ACLR and rehabilitation-only subgroups; these data are presented in Appendix 3 of the ESM. Three articles relating to the Delaware-Oslo ACL cohort had an overlapping participant cohort [32, 38, 39]. Additionally, one study [39] used participants that were also included in two other articles [33, 34]. This was managed by excluding one study [39] from the meta-analyses, as this study combined participants who were included in two other studies in the meta-analysis [34, 38].

### Risk of Bias

The two RCTs were rated with ROB2, with one study being rated overall as ‘low’ [9, 10], and the other as ‘serious’ risk of bias [31] largely because of concerns regarding a lack of ethics approval, and a lack of information about recruitment or a flow chart (Appendix 4 of the ESM). The remaining 13 studies (15 articles) were assessed with ROBINS-I (Appendix 5 of the ESM). Two studies [26, 30] were rated moderate risk of bias, three studies [33, 34, 38, 40] rated serious risk of bias and eight studies [27, 32, 35–37, 39, 41–43] rated critical risk of bias overall. Eight of the study designs specifically had a critical risk of confounding bias because of (a) advising patients in the rehabilitation-alone group not to return to sport or to modify their activities, in contrast to the advice given to ACLR patients and/or (b) allocating patients to the rehabilitation-alone group because they had no desire to return to sport or the treating staff felt their sporting aspirations did not warrant surgery. Both confounders would likely lead to the ACLR group being favoured for return to sport and physical activity participation outcomes. These eight studies formed the “high risk of confounding bias” sub-group in the meta-analyses.

### Return to Sport

#### Return-to-Sport Rates

**Fig. 2:**
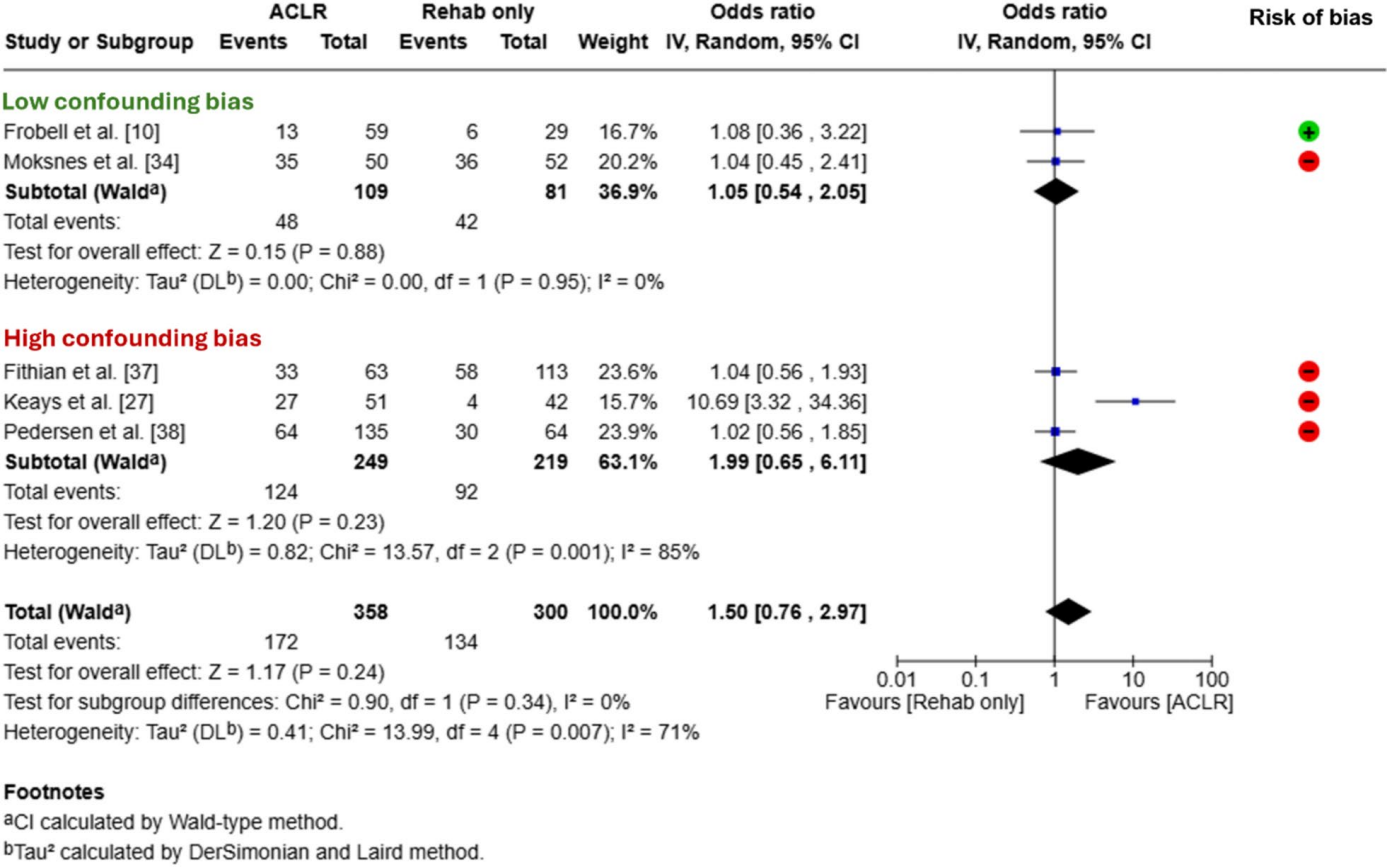
Random-effect meta-analysis depicting pooled odds of return to sport for all available studies, and according to the risk of confounding bias because of the study design. Studies were considered to have a high risk of confounding bias if they (a) recommended people with a low pre-injury activity level to choose non-surgical management, (b) allocated people to non-surgical management if they expressed they did not aim to return to pre-injury sport and/or (c) only advised people in the non-surgical group that they should limit activity or not return to sport. *ACLR* anterior cruciate ligament reconstruction, *CI* confidence interval, *IV* inverse variance, *Rehab* rehabilitation, ⊕ indicates an overall low risk of bias, ⊝ indicates an overall high risk of bias

Five studies reported the proportion of participants who returned to sport at 1–11 years after an ACL injury and were included in the meta-analyses [10, 27, 27, 34, 38]. All five studies assessed whether participants had returned to pre-injury levels of sport based on the activity level at the time of follow-up. To assess this, two studies used Tegner Activity Scale scores [10, 37], two studies used an activity-level classification (four levels of activity; level 1 = cutting/pivoting/jumping sports) [34, 38] and one study used a 6-point scale (where 0 = no sport, 5 = vigorous pivoting team sports at a competitive level) [27]. Overall, 172 out of 358 (48%) people in the ACLR groups had returned to sport, compared with 134 out of 300 (45%) in the rehabilitation-alone groups. The meta-analysis found a similar odds of returning to sport, irrespective of treatment with ACLR or rehabilitation alone (odds ratio 1.5, 95% CI 0.76–2.97, Fig. 2). Certainty of evidence was very low (Table 1). One study with a high risk of confounding bias reported a second measure of return-to-sport rate (not included in the meta-analysis) and found no difference in rates of return to any sport (ACLR 96%, rehabilitation alone 93%) [27]. In the sub-group meta-analysis, there were no differences in the odds of returning to sport between treatment groups for the two studies with a low risk of confounding bias [10, 34] (44% had returned to sport after ACLR and 52% had returned after rehabilitation alone, odds ratio 1.05, 95% CI 0.54–2.05, Fig. 2, low certainty, Table 1) or the three studies with a high risk of confounding bias [27, 37, 38] (50% had returned to sport after ACLR and 42% had returned after rehabilitation alone, odds ratio 1.99, 95% CI 0.65–6.11, Fig. 2). There was no difference in the odds of returning to sport at 1–4 years (two studies [9, 34], 1.16, 95% CI 0.64–2.10), or 5–10 years (four studies [10, 27, 37, 38], 1.70, 95% CI 0.71–4.04) follow-up (Appendix 6 of the ESM).

**Table 1:** GRADE (grading of recommendations, assessment, development and evaluations) assessment of certainty of evidence

			Certainty assessment						Number of participants in total (in meta-analysis		Effect	Certainty
Outcome	Number of studies	Study design	Risk of bias	Inconsistency^a^	Indirectness^b^	Imprecision^c^	Publication bias	Other considerations	ACLR	Rehab alone	Estimate (95% CI)	
Return-to-sport rate	5 (658)	1 RCT 4 non-randomised controlled trial	Very serious	Very serious *I*^2^ 71%	None	Serious	N/A	One additional result indicated no difference	(358)	(300)	No difference Odds ratio 1.5 (95% CI 0.76–2.97)	⨁◯◯◯ Very low
*Return-to sport rate (studies with a low risk of confounding bias)*	*2 (190)*	*1 RCT* *1 non-RCT*	*Serious*	*None* *I*^*2*^* 0%*	*None*	*Serious*	*N/A*		*(109)*	*(81)*	*No difference* *Odds ratio 1.05 (95%CI 0.54 to 2.05)*	*⨁⨁◯◯* *Low*
Time to return to sport	1 (82)	Retrospective matched pairs cohort study	Serious	None	None	Serious	N/A		43	39	No difference ACLR: mean 12 [10–16] months; Rehabilitation alone: 13 [10–17] months *P* = 0.375	⨁⨁◯◯ Low
Activity participation	13 (1280)	2 RCT 10 non-RCTs	Very serious	Very serious I^2^ 72%	None	Serious	Undetected	5 additional studies: 2 found a difference favouring ACLR [37, 40]; 3 found no difference between groups [32, 34, 38]	712 (318)	568 (255)	Small difference: mean difference in TAS 0.70 points (95% CI 0.16–1.24)	⨁◯◯◯ Very low
*Activity participation (studies with a low risk of confounding bias)*	*3 (418)*	*2 RCT* *1 non-RCT*	*Very serious*	*Very serious* *I*^*2*^* 73%*	*None*	*Very serious*	*N/A*	*1 additional study found no difference between groups *[34]	*232 (136)*	*186 (93)*	*No difference: mean difference in TAS 0.87 points (95% CI − 0.16 to 1.89)*	*⨁◯◯◯* *Very low*
Physical activity level	1 (70)	1 non-RCT	Very serious	-	None	Serious	N/A		33	37	No difference: approximate mean difference in IPAQ 355 (95% CI − 442 to 1152)	⨁◯◯◯ Very low

Publication bias was not assessed when ≤ 5 studies were available for a given outcome

*ACLR* anterior cruciate ligament reconstruction, *CI* confidence interval, *IPAQ* International Physical Activity Questionnaire, *N/A* not applicable, *RCT* randomised controlled trial, *TAS* Tegner Activity Scale

Certainty started at high and was downgraded to moderate (downgraded one level), low (downgraded two levels) or very low (downgraded three levels)

^a^None if *I*^2^ < 50%; serious if *I*^2^ 51–69%; very serious if *I*^2^ ≥ 70%

^b^Serious: some indirectness from review question; very serious: multiple indirectness

^c^Serious: < 400 participants or benefit/harm spans an effect size of 0.5 in either direction; very serious: < 400 participants and benefit/harm spans an effect size of 0.5 in both directions

#### Time to Return to Sport

Kovalak et al. [30] was the only study to evaluate time to return to sport. In this retrospective cohort study, match-paired treatment groups were formed based on sex, age, body mass index and activities and all patients were advised to avoid contact sports, irrespective of treatment strategy (moderate risk of bias). All patients returned to near their pre-injury level of physical activity after a similar time frame (ACLR group: mean 12 months; rehabilitation-alone group: mean 13 months). Certainty of evidence was low (Table 1).

### Activity Levels

#### Activity Participation Scales

**Fig. 3:**
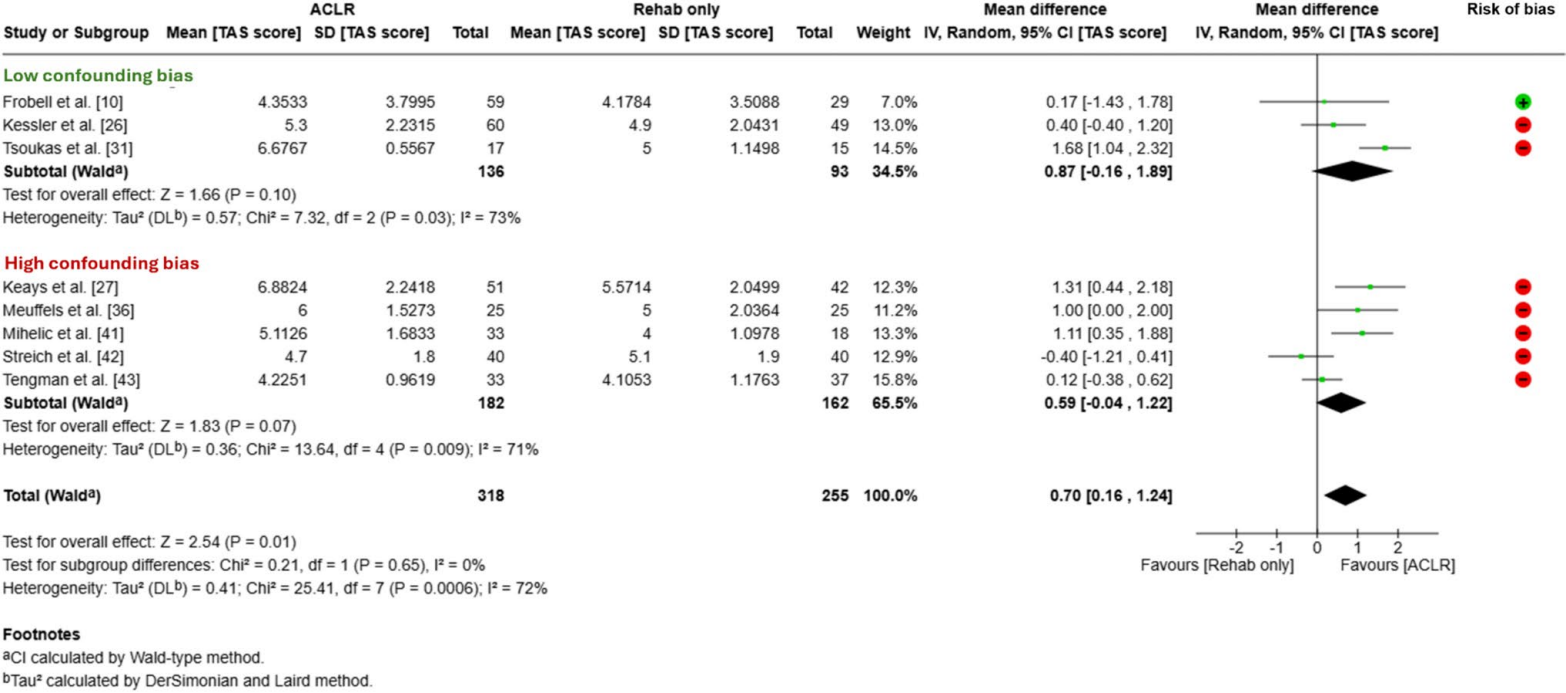
Random-effect meta-analysis depicting pooled mean difference (95% confidence interval [CI]) in the Tegner Activity Scale (TAS) score for all studies with data, separated according to the risk of confounding bias. Studies were considered to have a high risk of confounding bias if they (a) recommended people with a low pre-injury activity level to choose non-surgical management, (b) allocated people to non-surgical management if they expressed they did not aim to return to pre-injury sport and/or (c) only advised people in the non-surgical group that they should limit activity or not return to sport. *ACLR* anterior cruciate ligament reconstruction, *IV* inverse variance, *Rehab* rehabilitation, *SD* standard deviation, ⊕ indicates an overall low risk of bias; ⊝ indicates an overall high risk of bias

Tegner Activity Scale data from eight studies [10, 26, 27, 31, 36, 41, 42, 44] were pooled in the meta-analysis, with a total of 573 participants, and the follow-up ranged from a mean 5 [10] to 23 years [44] after ACL injury. A small difference was observed between groups, where ACLR groups had a pooled mean 0.70 points higher (95% CI 0.16–1.24) than groups managed with rehabilitation alone (Fig. 3). There was no between-group difference in self-reported activity participation in the sub-group of studies without a high risk of confounding bias (three studies [2, 7, 8], mean Tegner Activity Scale difference 0.87, 95% CI − 0.16 to 1.89) with a wide CI (Fig. 3). Tegner Activity Scale results from one study [37] could not be included in the meta-analysis because insufficient data to calculate SD were reported. Fithian et al. reported median Tegner Activity Scale scores were lower at the follow-up (mean 6.6 years after injury) in those managed without surgery (median 3) versus after early ACLR (median 6) [37]. In this study, treatment (i.e. group allocation) was influenced (rather than determined) by pre-injury activity level and other factors (critical risk of confounding bias probably favouring surgery).

Four studies used another method to assess activity participation [32, 34, 38, 40]. Moksnes et al. [34] (serious risk of bias) reported median activity participation categorised into four levels. They found no difference between groups: ACLR group median was 1 (95% CI 1–2) compared to the rehabilitation-alone group with a median of 2 (95% CI 1–2) [34]. Fink et al. [40] (serious risk of bias) categorised activity on a 0–3 scale with Level 3 representing high-risk pivoting sports. They found larger reductions in overall and in Level 3 sports participation in their rehabilitation-alone group compared with the ACLR group [40]. Pedersen et al. [38] (serious risk of bias) used the Marx Activity Rating Scale (range 0–16) and found no difference between groups (ACLR group mean 8, SD 4; rehabilitation-alone group mean 7, SD 4). Finally, in the Grindem et al. [32] study (critical risk of bias favouring ACLR group), participants self-rated activity participation based on sports (Levels 1–3) and frequency of participation per week. The crude (unadjusted) analysis suggested a group difference for Level 1 sports participation favouring ACLR (odds ratio for Level 1 sports participation 2.78, 95% CI 1.40–5.52) but not for Level 2 sports participation (odds ratio 0.65, 95% CI 0.37–1.14). However, an adjusted analysis was conducted where a propensity score was calculated based on baseline factors (propensity score was based on participation in Level 1 and Level 2 sport, sex, body mass index, concomitant injuries) to partially account for the confounding biases favouring the surgery group. The groups were then matched by propensity scores and there was no longer a between-group difference: Level 1 sports participating propensity score-adjusted odds ratio 1.3 (95% CI 0.61–2.78), and Level 2 sports participation propensity score-adjusted odds ratio 0.88 (95% CI 0.47–1.34), despite the rehabilitation-alone group being advised never to return to Level 1 sports. In summary, three out of four studies using a range of self-reported activity participation scales found no difference between groups. Certainty of evidence for activity participation was very low (Table 1).

A sub-group meta-analysis based on follow-up time found no difference in activity participation between groups for studies with follow-ups between 1 and 4 years after an ACL injury (one study [9], mean difference 0.08, 95% CI − 1.31 to 1.48) or studies with follow-ups 5–10 years after an ACL injury (three studies [10, 27, 31], mean difference 0.95, 95% CI − 0.09 to 1.99). A mean difference favouring ACLR groups was observed for studies with follow-ups of greater than 10 years post-ACL injury (six studies [26, 35, 36, 41, 42, 44], mean difference 0.65, 95% CI 0.02–1.28, all with a critical risk of confounding bias favouring surgery) [Appendix 7 of the ESM].

#### Overall Physical Activity Levels

One study included overall physical activity-level data. Tengman et al. [43] compared patients from one hospital who all underwent an ACLR (and were given a full return-to sport programme) with patients from another hospital who were all treated with rehabilitation alone (and were advised to modify activities and avoid certain sports). This study found no difference between groups in International Physical Activity Questionnaire scores (ACLR median 1563, range 480–7572; rehabilitation-alone median 1217, range 212–7398, very low certainty of evidence, Table 1).

## Discussion

This systematic review found no difference in return-to-sport or activity levels when people with ACL injury were managed with ACLR compared to rehabilitation alone. The certainty of this evidence was very low, mainly owing to the study designs predominantly having a high risk of bias. However, the bias due to confounders related to baseline differences and/or differences in the activity and return-to-sport advice given between groups was more likely to favour the ACLR groups and disadvantage the rehabilitation-alone groups. Specifically, ten of the 15 included studies either recommended non-surgical management if patients were less active pre-injury or did not plan to return to sport and/or only advised people in the non-surgical group that they should not return to sport. In many of the studies, ACLR was encouraged for those who expressed a desire to return to sport and only the people who chose ACLR were supported to return to sport. Our findings do not support the persistent beliefs that ACLR is required to return to cutting and pivoting sports, or that management with rehabilitation alone is not appropriate for individuals who desire participation in such sports.

### Return to Sport

The proportion of people who returned to a pre-injury level of sport after ACL injury was comparable irrespective of management with ACLR or rehabilitation alone. This does not align with a recent consensus statement informed by expert opinion, which recommends operative treatment as the preferred option for active patients wishing to return to jumping, cutting and pivoting sports [3]. Notably, the literature was only reviewed to find supporting evidence once consensus statements were finalised [3], which may explain why these recommendations are in contrast to the findings from this systematic review. The belief that ACLR is the best treatment option, or a requirement to return to sport, is widespread [45, 46] and reflected in online information, which rarely aligns with the best available evidence [47]. Inaccurate information could mislead patients and prevent them from making an informed treatment decision. Return to sport is a priority for many people who injure their ACL, with as many as 91% of people expecting to return to the same level of sport after an ACL injury [1]. Expectations and pre-conceived ideas about ACL injury treatment outcomes have potential for psychological impacts including a fear of re-injury that could affect the likelihood of returning to sport and future activity participation [48]. Considering the importance of return to sport to patients, including a strong relationship between return to sport and long-term quality of life [4], clinicians should provide evidence-based information to patients about their comparable likelihood of returning to sport irrespective of management with ACLR or rehabilitation alone. A previous meta-analysis of 69 articles found that 65% of people returned to pre-injury sport after ACLR [2]. Our findings suggest this proportion may be similar following management with rehabilitation alone, and patients should be informed that not everyone will return to their pre-injury sport after ACL injury, regardless of their treatment decision. The findings from this systematic review suggest that a desire to return to sport should not be a factor in recommending ACLR, as people can return to sport with either management option. The evidence suggests the likelihood of returning to sport is not as dependent on surgery as previously thought. Patients should be informed of the advantages and disadvantages of both surgical and non-surgical treatment options, so they can make an informed decision that aligns with their values and preferences. The type of clinician that a person with ACL injury consults first (e.g. consulting an orthopaedic surgeon first) for their ACL injury can influence the advice received and the likelihood of being recommended ACLR [45].

Only one study compared time to return to sport between groups. This study found people returned at a similar time post-injury between ACLR and rehabilitation-alone groups. However, patients should be aware that if they commence rehabilitation and decide at a later timepoint to have ACLR, their total time before return to sport will be prolonged.

A number of systematic reviews have investigated factors associated with the likelihood of returning to sport after ACLR [14, 49, 50]. Greater psychological readiness to return to sport, reduced kinesiophobia, higher athletic confidence, and greater preoperative knee self-efficacy and self-motivation have been associated with an increased likelihood of returning to sport after ACLR [14, 50]. It is possible that participants in some of the studies who were recommended ACLR because they wanted to return to sport differed in these psychological characteristics compared with those who were recommended rehabilitation alone because they did not want to return to sport. Furthermore, providing participants with different return-to-sport advice depending on the treatment group could impact on their knee confidence and kinesiophobia. Physical factors have also been associated with the return to sport, including limb symmetry [51], hop performance [2] and quadriceps strength [50]. It is not known whether the physical characteristics of participants at baseline differed between treatment groups. Additionally, the quality of rehabilitation within each study could influence both physical and psychological outcomes, and overall return-to-sport rates. Younger age may also be associated with a greater likelihood of returning to sport [2, 49]. Several studies reported a higher mean age in people managed with rehabilitation alone compared with the group treated with ACLR [27, 31, 35, 38, 43]. Differences in age between treatment groups could impact return-to-sport rates. Randomised controlled trials with return to sport as a primary outcome are needed to account for potential between-group differences in participant characteristics.

When informing patients about the advantages and disadvantages of each treatment option, a key consideration is the risk of experiencing an additional knee injury if they return to pre-injury sport. Reducing the risk of an additional knee injury should be a priority, as this can result in poor long-term outcomes including a higher risk of knee osteoarthritis, persistent pain and symptoms, and poorer quality of life [8]. There is a common belief that returning to cutting and pivoting sports after an ACL injury without surgery places the knee at a greater risk of secondary injury [3]. However, a recent systematic review could not determine if the incidence of new meniscal injuries is lower if an ACL injury is treated with surgery compared with treatment with rehabilitation only, owing to uncertainty in the evidence and serious bias in existing studies [52]. After this review was published, a Swedish pragmatic cohort study found that 11% of people managed with ACLR experienced a new knee injury within 2 years, compared with 6% of people after management with rehabilitation alone [53]. However, it is known that some individuals managed non-surgically will not regain the knee stability required to take part in cutting and pivoting sports [9, 11]. For individuals with persistent knee instability after trialling non-surgical management, ACL reconstruction can be recommended [54]. The risk of an ACL graft rupture is a further consideration for people managed with ACLR. Data suggest around one in five people have a second ACL injury in either knee after ACLR surgery [55–57], and this risk is higher for young people who return to sport [57]. Assessment of the impacts of returning to sport following management with ACLR or rehabilitation alone was outside the scope of this systematic review, but warrants further research. Additionally, recent research suggests that ACL ruptures can regain ligament continuity (as observed on magnetic resonance imaging) when managed with rehabilitation alone [58, 59]. This is an emerging area of research, and the relationship between regaining ligament continuity, return to sport and re-rupture rates has not been investigated.

Our search identified an additional RCT that compared the proportion of people returning to pre-injury sport after either early ACLR or initial management with rehabilitation and optional delayed ACLR [11]. This trial was excluded from our review because it only reported combined outcomes for people managed with rehabilitation alone and delayed ACLR. By the 2-year follow-up, 43% of the early ACLR group had returned to sport, compared with 31% of the initial rehabilitation and the optional delayed ACLR group. This is similar to the proportions who had returned to sport at 2 years in the KANON trial: 39% after management with initial rehabilitation and optional delayed ACLR, versus 44% who were randomised to early ACLR [9]. Findings are more difficult to interpret when participants who underwent delayed ACLR within 12 months prior to the primary endpoint are included together with rehabilitation-alone participants, as people are often not recommended to attempt return to sport until 12 months after surgery. Rather than suggesting return-to-sport rates are higher after ACLR, these data may reflect the prolonged time to return to sport for people who undergo delayed ACLR.

### Activity Levels

The meta-analysis found a difference of 0.70 points on the Tegner Activity Scale between groups favouring surgery. This difference is smaller than the minimal detectable change of 1.0 point and is close to the standard error of measurement (0.4–0.64) for this instrument [60]. In addition, findings from only two of five studies excluded from the meta-analysis favoured surgery, and excluding studies from the meta-analysis with a high risk of confounding biases likely to favour surgery found no difference in activity levels between groups (very low certainty of evidence). The Tegner Activity Scale considers the level of competition (where a higher score is given to people competing at a higher level) and demands of the sport or activity (where contact and pivoting/cutting sports receive higher scores than lower impact activities). It is surprising that even when participants in multiple studies were advised not to return to cutting or pivoting sports without surgery, and participants in several studies were managed with rehabilitation because they had a lower baseline activity level, we observed no meaningful difference in the activity level between treatment groups at 2–23 years after ACL injury.

Overall, most studies showed a reduction in activity participation across both ACLR and rehabilitation-alone groups. Importantly, the rehabilitation-alone groups in studies that were explicitly told to reduce their activity or not to return to sport often regained quite high levels of sports participation, equivalent to the ACLR groups in some studies.

### Strengths and Limitations

Strengths of this systematic review include a systematic and sensitive search, the consideration of confounding bias in the interpretation of findings and only including studies where exercise-based rehabilitation was standardised and/or supervised by a clinician. Interpretation of previous reviews comparing outcomes between strategies is often limited by inclusion of ‘non-surgical’ groups where the intervention is not described.

Several limitations of the evidence included in this review, and of the review process itself, warrant mention. The measure used by most studies to assess activity participation was the Tegner Activity Scale. This measure has some limitations. Notably, we cannot determine the frequency of sport participation, and the initial activity selection was by orthopaedic surgeons (with patient input later regarding the difficulty of these selected activities), meaning content validity cannot necessarily be assured [61, 62]. It is recommended, based on an expert consensus, to measure sport resumption and frequency after knee injury [13]. We would also recommend assessing *both* the highest level of sport that someone returned to at any timepoint post-injury, in addition to the level of sport that someone is participating in at a specific timepoint post-injury. Another consideration is whether a patient perceives themselves as having returned to their pre-injury level of sporting performance. Notably, there is no agreed gold standard measure for assessing these outcomes. Development of a new tool to measure return-to-sport outcomes after ACL injury, with robust psychometric properties, is warranted. The overall impact of confounding bias in favour of the ACLR groups may be underestimated, as a number of studies did not report the specific return-to-sport advice provided to patients in each arm. Four studies included in the meta-analysis reported data as median and range or interquartile range. These data were converted to means and SDs, which may introduce errors; however, errors were not expected to lead to bias favouring either group. Although the approach we used to convert median scores to mean scores is recommended in the *Cochrane Handbook* [63] and performs better than other methods [24], the approach assumes normally distributed outcomes that cannot be determined as this information was not reported within the articles. Despite this, it has been observed that the method we used also performs well when analysing non-normally distributed outcomes [64]. As there is no gold-standard method for assessing return to sport, three different measures were used to assess whether participants had returned to a pre-injury level of sport. This could have contributed to observed differences between studies. All studies included in the meta-analysis used the current sporting level at the time of follow-up to assess return to sport. Although it is an advantage that all studies included in the meta-analysis used this approach, assessing the highest level of sport that participants had returned to at any timepoint since ACL injury could have yielded different results, and warrants further research. Although we had planned on conducting a sub-group analysis to compare outcomes between delayed ACLR groups and rehabilitation-alone groups, we did not perform this analysis because of the long mean time between injury and surgery in some studies. It became apparent that we could not determine in some studies whether some participants commenced rehabilitation and underwent delayed ACLR, or whether this reflected long wait times for surgery in some countries. It is possible that by analysing people managed with rehabilitation alone we are biasing the sample towards people who have a successful non-surgical outcome, as those with an unsuccessful outcome may be most likely to undergo delayed ACLR. Although possible, this is not supported by data from the KANON trial, whereby similar return-to-sport rates and Tegner Activity Scale scores were observed between early ACLR, delayed ACLR and rehabilitation-alone treatment subgroups at 2 and 5 years following ACL injury [9, 10].

Further research is needed to compare return to sport and activity levels between early ACLR, delayed ACLR and rehabilitation-alone groups. Additionally, it is possible that outcomes differ within specific subgroups of participants, such as those with or without concomitant injuries, elite athletes compared to non-elite sport participants, or between younger and older participants; however, this could not be assessed within this systematic review. Studies included mostly adult participants and no studies included only elite athletes, so the findings cannot be generalised to children and adolescents, or elite athletes with ACL injury.

## Conclusions

The proportion of people with an ACL injury who returned to sport and the level of activity that they returned to was similar, irrespective of management with ACLR or rehabilitation alone. This is despite most study designs having a high risk of confounding biases likely favouring the ACLR participants. This evidence may be used to assist patients with ACL injuries making an informed treatment decision.

## Supplementary Information

Below is the link to the electronic supplementary material.

Supplementary file1 (PDF 176 KB)

Supplementary file2 (PDF 135 KB)

Supplementary file3 (PDF 48 KB)

Supplementary file4 (PDF 46 KB)

Supplementary file5 (PDF 67 KB)

Supplementary file6 (PDF 473 KB)

Supplementary file7 (PDF 332 KB)

## Declarations

### Availability of data and material

All data generated or analysed during this study are included in this published article and its supplementary information files.
