# Supplementary material for: No Difference in Return-to-Sport Rate or Activity Level in People with Anterior Cruciate Ligament (ACL) Injury Managed with ACL Reconstruction or Rehabilitation Alone: A Systematic Review and Meta-Analysis
Source: Sports Med. 2025 Jul 2;55(9):2191–205. doi: 10.1007/s40279-025-02268-5 (PMC12476414; doi:10.1007/s40279-025-02268-5)
Supplement: Supplementary file 1 — Supplementary file1 (PDF 176 KB) [file 40279_2025_2268_MOESM1_ESM.pdf]

Supplementary Appendix 1

1. **Search Strategy**
2. **Articles excluded at full text**

1. Search Strategy

MEDLINE

Ovid MEDLINE(R) and Epub Ahead of Print, In-Process, In-Data-Review & Other Non-Indexed Citations and Daily <1946 to July 20, 2023>

1	exp Anterior Cruciate Ligament/ or exp Anterior Cruciate Ligament Reconstruction/ or exp Anterior Cruciate Ligament Injuries/
2	(ACL* or anterior cruciate ligament*).tw,kf.
3	1 or 2
4	exp Return to Sport/ or exp Sports/
5	(activit* or (return* adj5 sport*) or (sport* adj5 level) or (sport* adj5 play*) or (return* adj5 play) or (return* adj5 exercis*) or (resum* adj5 sport*) or (sport* adj5 participa*) or RTS or (sport* adj5 level) or (resum* adj5 exercis*)).tw,kf.
6	(actigraph* or acceleromet* or "step count" or IPAQ* or MVPA or METS or "physical activity" or "International Physical Activity Questionnaire*" or Tegner or Marx or UCLA).tw,kf.
7	4 or 5 or 6
8	exp Exercise/ or exp conservative treatment/ or exp exercise therapy/ or exp rehabilitation/
9	(non-operat* or nonoperat* or "no* operati*" or "no reconstruction" or "no anterior cruciate ligament reconstruction" or "no ACL reconstruction" or copers or "ACL deficient" or conservative or non-surg* or "no* surg*" or rehab* or "exercise therapy" or "physi* supervised" or physiotherapy or "physical therapy").tw,kf.

10	8 or 9
11	3 and 7 and 10
12	Animals/
13	11 not 12

EMBASE

Embase Classic+Embase <1947 to July 20, 2023>

1	exp anterior cruciate ligament rupture/ or exp anterior cruciate ligament reconstruction/ or exp anterior cruciate ligament injury/ or exp anterior cruciate ligament/
2	(ACL* or anterior cruciate ligament*).tw,kf.
3	1 or 2
4	exp return to sport/ or exp sport/
5	(activit* or (return* adj5 sport*) or (sport* adj5 level) or (sport* adj5 play*) or (return* adj5 play) or (return* adj5 exercis*) or (resum* adj5 sport*) or (sport* adj5 participa*) or RTS or (sport* adj5 level) or (resum* adj5 exercis*)).tw,kf.
6	(actigraph* or acceleromet* or "step count" or IPAQ* or MVPA or METS or "physical activity" or "International Physical Activity Questionnaire*" or Tegner or Marx or UCLA).tw,kf.
7	4 or 5 or 6
8	exp kinesiotherapy/ or exp exercise/ or exp conservative treatment/ or exp rehabilitation/
9	(non-operat* or nonoperat* or "no* operati*" or "no reconstruction" or "no anterior cruciate ligament reconstruction" or "no ACL reconstruction" or copers or "ACL deficient" or conservative or non-surg* or "no* surg*" or rehab* or "exercise therapy" or "physi* supervised" or physiotherapy or "physical therapy").tw,kf.
10	8 or 9

11	3 and 7 and 10
12	animal/
13	11 not 12

CINAHL – updated and run July 20, 2023

#	Query
S6	S4 NOT S5
S5	SU (animal)
S4	S1 AND S2 AND S3
S3	TI (non-operat* or nonoperat* or "no* operati*" or "no reconstruction" or "no anterior cruciate ligament reconstruction" or "no ACL reconstruction" or copers or "ACL deficient" or conservative or non-surg* or "no* surg*" or rehab* or "exercise therapy" or "physi* supervised" or physiotherapy or "physical therapy") OR AB (non-operat* or nonoperat* or "no* operati*" or "no reconstruction" or "no anterior cruciate ligament reconstruction" or "no ACL reconstruction" or copers or "ACL deficient" or conservative or non-surg* or "no* surg*" or rehab* or "exercise therapy" or "physi* supervised" or physiotherapy or "physical therapy") OR (non-operat* or nonoperat* or "no* operati*" or "no reconstruction" or "no anterior cruciate ligament reconstruction" or "no ACL reconstruction" or copers or "ACL deficient" or conservative or non-surg* or "no* surg*" or rehab* or "exercise therapy" or "physi* supervised" or physiotherapy or "physical therapy") OR SU (exercise or physical therapy or rehabilitation) OR SU (exercise or physical therapy or rehabilitation)
S2	TI ("return to sport" or "return to activity" or "return to performance" or "return to play" or "return to physical activity" or actigraph* or acceleromet* or "step count" or IPAQ* or MVPA or METS or "physical activity" or "International Physical Activity Questionnaire*" or Tegner or Marx or UCLA) OR AB ("return to sport" or "return to activity" or "return to performance" or "return to play" or "return to physical activity" or actigraph* or acceleromet* or "step count" or IPAQ* or MVPA or METS or "physical activity" or "International Physical Activity Questionnaire*" or Tegner or Marx or UCLA) OR ("return to sport" or "return to activity" or "return to performance" or "return to play" or "return to physical activity" or actigraph* or acceleromet* or "step count" or IPAQ* or MVPA or METS or "physical activity" or "International Physical Activity Questionnaire*" or Tegner or Marx or UCLA) OR SU (“sports re-entry”)
S1	TI (ACL* or "anterior cruciate ligament*") OR AB (ACL* or "anterior cruciate ligament*") OR (ACL* or "anterior cruciate ligament*") OR SU ("Anterior cruciate ligament injuries")

SPORTDiscus – updated and run July 20, 2023

#	Query
S6	S4 NOT S5
S5	TI animal* OR AB animal* OR animal*
S4	S1 AND S2 AND S3
S3	TI (non-operat* or nonoperat* or "no* operati*" or "no reconstruction" or "no anterior cruciate ligament reconstruction" or "no ACL reconstruction" or copers or "ACL deficient" or conservative or non-surg* or "no* surg*" or rehab* or "exercise therapy" or "physi* supervised" or physiotherapy or "physical therapy") OR AB (non-operat* or nonoperat* or "no* operati*" or "no reconstruction" or "no anterior cruciate ligament reconstruction" or "no ACL reconstruction" or copers or "ACL deficient" or conservative or non-surg* or "no* surg*" or rehab* or "exercise therapy" or "physi* supervised" or physiotherapy or "physical therapy") OR (non-operat* or nonoperat* or "no* operati*" or "no reconstruction" or "no anterior cruciate ligament reconstruction" or "no ACL reconstruction" or copers or "ACL deficient" or conservative or non-surg* or "no* surg*" or rehab* or "exercise therapy" or "physi* supervised" or physiotherapy or "physical therapy") OR SU (“physical therapy” or rehabilitation or exercise)
S2	TI ("return to sport" or "return to activity" or "return to performance" or "return to play" or "return to physical activity" or actigraph* or acceleromet* or "step count" or IPAQ* or MVPA or METS or "physical activity" or "International Physical Activity Questionnaire*" or Tegner or Marx or UCLA) OR AB ("return to sport" or "return to activity" or "return to performance" or "return to play" or "return to physical activity" or actigraph* or acceleromet* or "step count" or IPAQ* or MVPA or METS or "physical activity" or "International Physical Activity Questionnaire*" or Tegner or Marx or UCLA) OR ("return to sport" or "return to activity" or "return to performance" or "return to play" or "return to physical activity" or actigraph* or acceleromet* or "step count" or IPAQ* or MVPA or METS or "physical activity" or "International Physical Activity Questionnaire*" or Tegner or Marx or UCLA) OR SU (sports)
S1	TI (ACL* or "anterior cruciate ligament*") OR AB (ACL* or "anterior cruciate ligament*") OR (ACL* or "anterior cruciate ligament*") OR SU (“Anterior cruciate ligament” or “anterior cruciate ligament surgery” or “anterior cruciate ligament injuries”)

COCHRANE CENTRAL REGISTER last run July 20, 2023

ID	Search
#1	MeSH descriptor: [Anterior Cruciate Ligament] explode all trees
#2	MeSH descriptor: [Anterior Cruciate Ligament Injuries] explode all trees
#3	MeSH descriptor: [Anterior Cruciate Ligament Reconstruction] explode all trees

#4	((anterior near/2 cruciate* near/2 ligament*) or acl):ti,ab,kw (Word variations have been searched)
#5	#1 or #2 or #3 or #4
#6	MeSH descriptor: [Return to Sport] explode all trees
#7	MeSH descriptor: [Sports] explode all trees
#8	(activit* or "return* near/5 sport*" or "sport* near/5 level" or "sport* near/5 play*" or "return* near/5 play" or "return* near/5 exercis*" or "resum* near/5 sport*" or "sport* near/5 participa*" or RTS or "sport* near/5 level" or "resum* near/5 exercis*"):ti,ab,kw (Word variations have been searched)
#9	(actigraph* or acceleromet* or "step count" or IPAQ* or MVPA or METS or "physical activity" or "International Physical Activity Questionnaire" or Tegner or Marx or UCLA):ti,ab,kw (Word variations have been searched)
#10	#6 or #7 or #8 or #9
#11	MeSH descriptor: [Exercise] explode all trees
#12	MeSH descriptor: [Conservative Treatment] explode all trees
#13	MeSH descriptor: [Exercise Therapy] explode all trees
#14	MeSH descriptor: [Rehabilitation] explode all trees
#15	(non-operat* or nonoperat* or "no* operati*" or "no reconstruction or "no anterior cruciate ligament reconstruction" or "no ACL reconstruction" or copers or "ACL deficient" or conservative or non-surg* or "no* surg" or rehab* or "exercise therapy" or physi* supervised" or physiotherapy or "physical therapy"):ti,ab,kw (Word variations have been searched)
#16	#11 or #12 or #13 or #14 or #15
#17	#5 and #10 and #16
#18	(animal*):ti,ab,kw (Word variations have been searched)
#19	#17 not #18

2. Articles Excluded at Full Text Screening

#	Authors; Title; Published Year; Journal; Volume; Issue; Pages; DOI	Reason for exclusion
1	Ageberg, Eva; Thomee, Roland; Neeter, Camille; Silbernagel, Karin Gravare; Roos, Ewa M; Muscle strength and functional performance in patients with anterior cruciate ligament injury treated with training and surgical reconstruction or training only: a two to five-year followup.; 2008; Arthritis and rheumatism; 59; 12; 1773-9; https://dx.doi.org/10.1002/art.24066	Wrong outcomes
2	Andersson C.; Odensten M.; Gillquist J. ; Knee function after surgical or nonsurgical treatment of acute rupture of the anterior cruciate ligament: A randomized study with a long-term follow-up period; 1991; Clinical Orthopaedics and Related Research; 264; (Andersson, Odensten, Gillquist) Dept. of Orthopaedic Surgery, University Hospital, S-581 85 Linkoping, Sweden; 255-263	Wrong intervention
3	Andersson, C; Gillquist, J; Treatment of acute isolated and combined ruptures of the anterior cruciate ligament. A long-term follow-up study.; 1992; The American journal of sports medicine; 20; 1; 7-Dec;	Wrong intervention
4	Andersson, C; Odensten, M; Good, L; Gillquist, J; Surgical or non-surgical treatment of acute rupture of the anterior cruciate ligament. A randomized study with long-term follow-up.; 1989; The Journal of bone and joint surgery. American volume; 71; 7; 965-74	Wrong intervention
5	Baltaci G.; Ergun N.; Bayrakci V. ; Non-operative treatment of anterior cruciate ligament injuries; 1997; Sports Excercise and Injury; 3; 4; 160-163	Wrong study design
6	Blanke, F.; Trinnes, K.; Oehler, N.; Prall, W. C.; Lutter, C.; Tischer, T.; Vogt, S.; Spontaneous healing of acute ACL ruptures: rate, prognostic factors and short-term outcome; 2023; Archives of Orthopaedic and Trauma Surgery; 143(7); ; 4291-4298; https://dx.doi.org/10.1007/s00402-022-04701-0	Wrong study design
7	Bryant A.L.; Clark R.A.; Pua Y.-H. ; Morphology of hamstring torque-time curves following acl injury and reconstruction: Mechanisms and implications; 2011; Journal of Orthopaedic Research; 29; 6; 907-914; http://dx.doi.org/10.1002/jor.21306	Wrong outcomes
8	Bryant, A.L.; Creaby, M.W.; Newton, R.U.; Steele, J.R.; Dynamic Restraint Capacity of the Hamstring Muscles Has Important Functional Implications After Anterior Cruciate Ligament Injury and Anterior Cruciate Ligament Reconstruction; 2008; Archives of Physical Medicine and Rehabilitation; 89; 12; 2324-2331; 10.1016/j.apmr.2008.04.027	Wrong outcomes
9	Button, Kate; Roos, Paulien E; van Deursen, Robert W M; Activity progression for anterior cruciate ligament injured individuals.; 2014; Clinical biomechanics (Bristol, Avon); 29; 2; 206-12; https://dx.doi.org/10.1016/j.clinbiomech.2013.11.010	Wrong comparator
10	Casteleyn, P P; Handelberg, F; Non-operative management of anterior cruciate ligament injuries in the general population.; 1996; The Journal of bone and joint surgery. British volume; 78; 3; 446-51	Wrong study design
11	Costa-Paz M.; Garcia-Mansilla I.; Ayerza M.; Muscolo D.L. ; Spontaneous healing in complete ACL ruptures: Results at eight-year mean follow-up; 2018; Orthopaedic Journal of Sports Medicine; 6; 12 Supplement 5; http://dx.doi.org/10.1177/2325967118S00188	Conference abstract
12	Dahlstedt L.; Dalen N. ; Outcome of patients with anterior cruciate ligament injuries selected for conservative treatment; 1991; Scandinavian Journal of Medicine and Science in Sports; 1; 4; 239-243	Wrong intervention
13	Daniel, D M; Stone, M L; Dobson, B E; Fithian, D C; Rossman, D J; Kaufman, K R; Fate of the ACL-injured patient. A prospective outcome study.; 1994; The American journal of sports medicine; 22; 5; 632-44	Wrong comparator

14	Davies, Loretta; Cook, Jonathan; Leal, Jose; Areia, Carlos Morgado; Shirkey, Beverly; Jackson, William; Campbell, Helen; Fletcher, Heidi; Carr, Andrew; Barker, Karen; Lamb, Sarah E; Monk, Paul; O'Leary, Sean; Haddad, Fares; Wilson, Chris; Price, Andrew; Beard, David; Comparison of the clinical and cost effectiveness of two management strategies (rehabilitation versus surgical reconstruction) for non-acute anterior cruciate ligament (ACL) injury: study protocol for the ACL SNNAP randomised controlled trial.; 2020; Trials; 21; 1; 405; https://dx.doi.org/10.1186/s13063-020-04298-y	Wrong study design
15	DePalma, B.F.; Zelko, R.R.; Knee rehabilitation following anterior cruciate ligament injury or surgery.; 1986; Athletic Training; 21; 3; 200-204	Wrong study design
16	Diekstall, P; Rauhut, F; [Considerations for the indications for anterior cruciate ligament reconstruction. Results of conservative versus operative treatment].; 1999; Der Unfallchirurg; 102; 3; 173-81	Study not in English
17	Diercks R.L.; Dijkstra M. ; Surgically and conservatively treated patients with post-traumatic anterior cruciate ligament insufficiency: Comparison of results; 2001; Geneeskunde en Sport; 34; 5; 175-179	Study not in English
18	Dobos, J.; Hehl, I.; Leopold, L.; Gyermek- es serdulokoru sportolok elulso keresztszalag szakadasanak kezelesi lehetosegei es azok eredmenyei osztalyunkon. / Possibilities and results in the treatment of ACL tears in children and adolescent athletes in our department.; 1996; Sportorvosi Szemle/Hungarian Review of Sports Medicine; 37; 3; 126-135	Study not in English
19	Dunn, Kristina L; Lam, Kenneth C; Valovich McLeod, Tamara C; Early Operative Versus Delayed or Nonoperative Treatment of Anterior Cruciate Ligament Injuries in Pediatric Patients.; 2016; Journal of athletic training; 51; 5; 425-7; https://dx.doi.org/10.4085/1062-6050.51.5.11	Wrong study design
20	Eastlack, M E; Axe, M J; Snyder-Mackler, L; Laxity, instability, and functional outcome after ACL injury: copers versus noncopers.; 1999; Medicine and science in sports and exercise; 31; 2; 210-5;	Wrong study design
21	Ehlinger, Matthieu; Panisset, Jean-Claude; Dejour, David; Gonzalez, Jean-Francois; Paihle, Regis; Favreau, Henri; Ollivier, Matthieu; Lustig, Sebastien; Francophone Arthroscopy Society (SFA); Anterior cruciate ligament reconstruction in the over-50s. A prospective comparative study between surgical and functional treatment.; 2021; Orthopaedics & traumatology, surgery & research : OTSR; 107; 8S; 103039; https://dx.doi.org/10.1016/j.otsr.2021.103039	Wrong comparator
22	Ekas, Guri Ranum; Moksnes, Havard; Grindem, Hege; Risberg, May Arna; Engebretsen, Lars; Coping With Anterior Cruciate Ligament Injury From Childhood to Maturation: A Prospective Case Series of 44 Patients With Mean 8 Years' Follow-up.; 2019; The American journal of sports medicine; 47; 1; 22-30; https://dx.doi.org/10.1177/0363546518810750	Wrong study design
23	Ericsson YB; Roos EM; Frobell RB; Lower extremity performance following ACL rehabilitation in the KANON-trial: impact of reconstruction and predictive value at 2 and 5 years [with consumer summary]; 2013; British Journal of Sports Medicine 2013 Oct; 47(15):980-985	Wrong outcomes
24	Faltstrom, Anne; Hagglund, Martin; Kvist, Joanna; Patient-reported knee function, quality of life, and activity level after bilateral anterior cruciate ligament injuries.; 2013; The American journal of sports medicine; 41; 12; 2805-13; https://dx.doi.org/10.1177/0363546513502309	Wrong study design
25	Fashkhami, A.N.; Rahimi, A.; Kalantari, K.K.; Baghban, A.A.; Naimi, S.S.; Keyhani, S.; The functional abilities and maximal vertical jumping height in coper and non-coper anterior cruciate ligament-deficient knee; 2014; Iranian Rehabilitation Journal; 12; 20; 28-33;	Wrong intervention
26	Feagin, J.A.; Curl, W.W.; Isolated tear of the anterior cruciate ligament: five-year follow-up study.; 1990; Journal of Orthopaedic & Sports Physical Therapy; 12; 6; 232-236	Wrong intervention

27	Filbay S.; Andersson C.; Ardern C.; Gauffin H.; Kvist J. ; Prognostic factors for knee pain, symptoms, function and quality of life 32-37 years after surgically or non-surgically managed ACL injury; 2019; Journal of Science and Medicine in Sport; 22; Supplement 2; S57; http://dx.doi.org/10.1016/j.jsams.2019.08.251	Wrong outcomes
28	Filbay S.R.; Andersson C.; Ardern C.; Gauffin H.; Kvist J. ; Patient-reported outcome 32 to 37 years following surgically treated or non-surgically treated acute anterior cruciate ligament injury; 2018; Osteoarthritis and Cartilage; 26; Supplement 1; S52-S53	Wrong intervention
29	Filbay S.R.; Ardern C.; Andersson C.; Gauffin H.; Kvist J. ; Prognostic factors for radiographic and symptomatic tibiofemoral and patellofemoral osteoarthritis 32-37 years after anterior cruciate ligament injury; 2020; Osteoarthritis and Cartilage; 28; Supplement 1; S350-S351; http://dx.doi.org/10.1016/j.joca.2020.02.548	Wrong outcomes
30	Filbay S.R.; Frobell R.B.; Lohmander S.; Roos E.M. ; Surgery, cartilage injury and patient-reported measures within 4 weeks of ACL rupture are associated with 5 year outcome: Exploratory analysis of the KANON trial; 2015; Osteoarthritis and Cartilage; 23; SUPPL. 2; A274-A275	Conference abstract
31	Filbay, S R; Culvenor, A G; Ackerman, I N; Russell, T G; Crossley, K M; Quality of life in anterior cruciate ligament-deficient individuals: a systematic review and meta-analysis.; 2015; British journal of sports medicine; 49; 16; 1033-41; https://dx.doi.org/10.1136/bjsports-2015-094864	Wrong study design
32	Filbay, S. R.; Dowsett, M.; Chaker Jomaa, M.; Rooney, J.; Sabharwal, R.; Lucas, P.; Van Den Heever, A.; Kazaglis, J.; Merlino, J.; Moran, M.; Allwright, M.; Kuah, D. E. K.; Durie, R.; Roger, G.; Cross, M.; Cross, T.; Healing of acute anterior cruciate ligament rupture on MRI and outcomes following non-surgical management with the Cross Bracing Protocol; 2023; BJSM online; 14; ; 14; https://dx.doi.org/10.1136/bjsports-2023-106931	Wrong study design
33	Filbay, S.; Dowsett, M.; Chaker Jomaa, M.; Rooney, J.; Kazaglis, J.; Merlino, J.; Moran, M.; Allwright, M.; Kuah, D.; Sabharwal, R.; van den Heever, A.; Lucas, P.; Roger, G.; Cross, M.; Cross, T.; Healing of a ruptured anterior cruciate ligament and patient-reported outcomes following non-surgical management with a novel bracing protocol; 2022; Journal of Science and Medicine in Sport; 25(Supplement 2); S29-S30; https://dx.doi.org/10.1016/j.jsams.2022.09.006	Conference abstract
34	Filbay, S.; Roemer, F.; Lohmander, S.; Turkiewicz, A.; Roos, E. M.; Frobell, R.; Englund, M.; SPONTANEOUS HEALING OF THE RUPTURED ANTERIOR CRUCIATE LIGAMENT: OBSERVATIONS FROM THE KANON TRIAL; 2022; BMJ open sport and exercise medicine; 8; ; A3; 10.1136/bmjsem-2022-sportskongres.8	Conference abstract
35	Filbay, S.; Roemer, F.; Lohmander, S.; Turkiewicz, A.; Roos, E. M.; Frobell, R.; Englund, M.; Spontaneous Healing of the Ruptured Anterior Cruciate Ligament: Observations from the Kanon Trial; 2022; BMJ Open Sport and Exercise Medicine; 8(Supplement 1); A3; https://dx.doi.org/10.1136/bmjsem-2022-sportskongres.8	Conference abstract
36	Filbay, S; Gauffin, H; Andersson, C; Kvist, J; Prognostic factors for tibiofemoral and patellofemoral osteoarthritis 32-37 years after anterior cruciate ligament injury managed with early surgical repair or rehabilitation alone.; 2021; Osteoarthritis and cartilage; 29; 12; 1682-1690; https://dx.doi.org/10.1016/j.joca.2021.08.009	Wrong intervention
37	Filbay, Stephanie R; Roos, Ewa M; Frobell, Richard B; Roemer, Frank; Ranstam, Jonas; Lohmander, L Stefan; Delaying ACL reconstruction and treating with exercise therapy alone may alter prognostic factors for 5-year outcome: an exploratory analysis of the KANON trial.; 2017; British journal of sports medicine; 51; 22; 1622-1629; https://dx.doi.org/10.1136/bjsports-2016-097124	Wrong outcomes
38	Filbay, Stephanie; Andersson, Christer; Gauffin, Hakan; Kvist, Joanna; Prognostic Factors for Patient-Reported Outcomes at 32 to 37 Years After Surgical or Nonsurgical Management of Anterior Cruciate Ligament Injury.; 2021; Orthopaedic journal of sports medicine; 9; 8; 2.33E+16; https://dx.doi.org/10.1177/23259671211021592	Wrong outcomes

39	Filbay, Stephanie; Kvist, Joanna; Fear of Reinjury Following Surgical and Nonsurgical Management of Anterior Cruciate Ligament Injury: An Exploratory Analysis of the NACOX Multicenter Longitudinal Cohort Study.; 2022; Physical therapy; 102; 2; ; https://dx.doi.org/10.1093/ptj/pzab273	Wrong outcomes
40	Fink C.; Hoser C.; Benedetto K.P. ; Development of osteoarthritis after anterior cruciate ligament injury. Operative versus conservative treatment; 1994; Unfallchirurg; 97; 7; 357-361	Study not in English
41	Fink C.; Hoser C.; Benedetto K.P.; Hackl W.; Gabl M. ; Long term results following conservative or operative treatment of anterior cruciate ligament rupture; 1996; Unfallchirurg; 99; 12; 964-969; http://dx.doi.org/10.1007/s001130050081	Study not in English
42	Fink, C.; Genelin, A.; Benedetto, K.P.; Hoser, C.; Sperner, G.; Treatment of acute ruptures of the knee cruciate ligament in relation to age and level of sport leisure ; 1994; SPORT Schweizerische Zeitschrift fur Medizin und Traumatologie; 1; 26-29;	Conference abstract
43	Fink, C; Genelin, A; Benedetto, K P; Hoser, C; Sperner, G; [The treatment of fresh anterior cruciate ligament ruptures in relation to age and level of sports activity].; 1994; Schweizerische Zeitschrift fur Medizin und Traumatologie = Revue suisse pour medecine et traumatologie; 1; 26-Sep;	Study not in English
44	Fink, C; Hoser, C; Benedetto, K P; [Development of arthrosis after rupture of the anterior cruciate ligament. A comparison of surgical and conservative therapy].; 1994; Der Unfallchirurg; 97; 7; 357-61	Study not in English
45	Fink, C; Hoser, C; Benedetto, K P; [Sports capacity after rupture of the anterior cruciate ligament--surgical versus non-surgical therapy].; 1993; Aktuelle Traumatologie; 23; 8; 371-5;	Study not in English
46	Fink, C; Hoser, C; Benedetto, K P; Hackl, W; Gabl, M; [Long-term outcome of conservative or surgical therapy of anterior cruciate ligament rupture].; 1996; Der Unfallchirurg; 99; 12; 964-9;	Study not in English
47	Fjellman-Wiklund A.; Soderman K.; Lundqvist M.; Hager C. ; How does an ACL injury influence life in the long term?-an interview study; 2015; Physiotherapy (United Kingdom); 101; SUPPL. 1; eS388-eS389; http://dx.doi.org/10.1016/j.physio.2015.03.611	Wrong study design
48	Flosadottir V.; Roos E.M.; Ageberg E. ; Muscle function at 3 years following ACL injury is associated with 5-year patient-reported outcomes; 2016; Osteoarthritis and Cartilage; 24; SUPPL. 1; S25-S26	Conference abstract
49	Flosadottir, Vala; Frobell, Richard; Roos, Ewa M; Ageberg, Eva; Impact of treatment strategy and physical performance on future knee-related self-efficacy in individuals with ACL injury.; 2018; BMC musculoskeletal disorders; 19; 1; 50; https://dx.doi.org/10.1186/s12891-018-1973-2	Wrong outcomes
50	Flosadottir, Vala; Roos, Ewa M; Ageberg, Eva; Muscle function is associated with future patient-reported outcomes in young adults with ACL injury.; 2016; BMJ open sport & exercise medicine; 2; 1; e000154	Wrong outcomes
51	Fowler P. ; Rehabilitation and early anterior cruciate ligament reconstruction was not better than rehabilitation and delayed reconstruction: Commentary; 2011; Journal of Bone and Joint Surgery - Series A; 93; 4; 395; http://dx.doi.org/10.2106/JBJS.9304.ebo579	Wrong study design
52	Fowler, P J; Regan, W D; The patient with symptomatic chronic anterior cruciate ligament insufficiency. Results of minimal arthroscopic surgery and rehabilitation.; 1987; The American journal of sports medicine; 15; 4; 321-5;	Wrong intervention
53	Fowler, P.J.; Regan, W.D.; The patient with symptomatic chronic anterior cruciate ligament insufficiency. Results of minimal arthroscopic surgery and rehabilitation. / Patient atteint d ' une insuffisance symptomatique chronique du ligament croise anterieur: resultats d ' une leger; 1987; American Journal of Sports Medicine; 15; 4; 321-325	Wrong comparator
54	Friden, T; Zatterstrom, R; Lindstrand, A; Moritz, U; Anterior-cruciate-insufficient knees treated with physiotherapy. A three-year follow-up study of patients with late diagnosis.; 1991; Clinical orthopaedics and related research; 263; 190-9;	Wrong study design

55	Frobell R. ; The outcome after acute ACL injury-is surgery always needed and what should the patient expect?; 2012; Annals of Physical and Rehabilitation Medicine; 55; SUPPL.1; e55; http://dx.doi.org/10.1016/j.rehab.2012.07.142	Duplicate
56	Frobell, R. B.; Roos, E. M.; Roos, H. P.; Ranstam, J.; Lohmander, L. S.; Reading Research: The ACL Tear- to Cut or Not to Cut.; 2010; Performance Conditioning Volleyball; 16; 4; 5-Jun;	Wrong study design
57	Gauffin H.; Filbay S.R.; Andersson C.; Ardern C.; Kvist J. ; Radiographic osteoarthritis and knee symptoms 32-37 years following acute anterior cruciate ligament injury; 2019; Osteoarthritis and Cartilage; 27; Supplement 1; S230-S231; http://dx.doi.org/10.1016/j.joca.2019.02.359	Wrong intervention
58	Gfoller, Peter; Abermann, Elisabeth; Runer, Armin; Hoser, Christian; Pfluglmayer, Mario; Wierer, Guido; Fink, Christian; Non-operative treatment of ACL injury is associated with opposing subjective and objective outcomes over 20 years of follow-up.; 2019; Knee surgery, sports traumatology, arthroscopy : official journal of the ESSKA; 27; 8; 2665-2671; https://dx.doi.org/10.1007/s00167-018-5296-5	Wrong study design
59	Gille, J.; Paech, A.; JÃ¼rgens, C.; Surgical and conservative treatment of anterior cruciate ligament rupture in sports ; 2016; Trauma und Berufskrankheit; 18; ; 506-510; 10.1007/s10039-016-0156-3	Wrong study design
60	Grevnerts, Hanna Tigerstrand; Sonesson, Sofi; Gauffin, Hakan; Ardern, Clare L.; Stalman, Anders; Kvist, Joanna; Decision Making for Treatment After ACL Injury From an Orthopaedic Surgeon and Patient Perspective.; 2021; Orthopaedic Journal of Sports Medicine; 9; 4; 1-Aug; 10.1177/23259671211005090	Wrong outcomes
61	Grindem, Hege; Wellsandt, Elizabeth; Failla, Mathew; Snyder-Mackler, Lynn; Risberg, May Arna; Anterior Cruciate Ligament Injury-Who Succeeds Without Reconstructive Surgery? The Delaware-Oslo ACL Cohort Study.; 2018; Orthopaedic journal of sports medicine; 6; 5; 2.33E+15; https://dx.doi.org/10.1177/2325967118774255	Wrong outcomes
62	Gupta, Ravi; Masih, Gladson David; Chander, Gaurav; Bachhal, Vikas; Delay in surgery predisposes to meniscal and chondral injuries in anterior cruciate ligament deficient knees.; 2016; Indian journal of orthopaedics; 50; 5; 492-498	Wrong outcomes
63	Hager C.; Brax-Olofsson L.; Tengman E.; Tegner Y.; Grip H.; Rydh A.; Stensdotter A.-K.; Lundgren L.; Nilsson K.G. ; Knee function after acl injury-a long term follow up addressing treatment, movement capacity, osteoarthritis and quality of life; 2011; Physiotherapy (United Kingdom); 97; SUPPL. 1; eS445; http://dx.doi.org/10.1016/j.physio.2011.04.002	Conference abstract
64	Harris, Kyle; Driban, Jeffrey Bradford; Sitler, Michael R; Cattano, Nicole M; Hootman, Jennifer M; Five-year clinical outcomes of a randomized trial of anterior cruciate ligament treatment strategies: an evidence-based practice paper.; 2015; Journal of athletic training; 50; 1; 110-2; https://dx.doi.org/10.4085/1062-6050-49.3.53	Wrong study design
65	Hartigan E; Knee function after ACL rupture and reconstruction effects of neuromuscular training.; 2009; Knee Function After Acl Rupture & Reconstruction Effects of Neuromuscular Training; Ph.D.; 200 p-200 p	Wrong study design
66	Hayes-Lattin, M.; Sylvia, S. M.; Bragg, J. T.; Puzzitiello, R. N.; Richmond, J. C.; Salzler, M. J.; Subjective Outcomes After Allograft Reconstruction and Nonoperative Treatment of Anterior Cruciate Ligament Ruptures Are Similar in Patients Aged 40 Years and Older: A 2:1 Propensity Score-Matched Analysis; 2023; Arthrosc Sports Med Rehabil; 5; 3; e657-e662; https://dx.doi.org/10.1016/j.asmr.2023.03.005	Wrong intervention
67	Hayes-Lattin, M.; Sylvia, S. M.; Bragg, J. T.; Puzzitiello, R. N.; Richmond, J. C.; Salzler, M. J.; Subjective Outcomes After Allograft Reconstruction and Nonoperative Treatment of Anterior Cruciate Ligament Ruptures Are Similar in Patients Aged 40 Years and Older: A 2:1 Propensity Score-Matched Analysis; 2023; Arthroscopy, Sports Medicine, and Rehabilitation; 5(3); ; e657-e662; https://dx.doi.org/10.1016/j.asmr.2023.03.005	Wrong intervention

68	Hetsroni, Iftach; Delos, Demetris; Fives, Greg; Boyle, Brian W; Lillemoe, Kaitlyn; Marx, Robert G; Nonoperative treatment for anterior cruciate ligament injury in recreational alpine skiers.; 2013; Knee surgery, sports traumatology, arthroscopy : official journal of the ESSKA; 21; 8; 1910-4; https://dx.doi.org/10.1007/s00167-012-2324-8	Wrong intervention
69	Hoogeslag, R. A. G.; Huis In 't Veld, R.; Brouwer, R. W.; de Graaff, F.; Verdonschot, N.; Acute Anterior Cruciate Ligament Rupture: repair or Reconstruction? Five-Year Results of a Randomized Controlled Clinical Trial; 2022; American journal of sports medicine; 50; 7; 1779â€ 1787; 10.1177/03635465221090527	Wrong intervention
70	Hurd, Wendy J; Axe, Michael J; Snyder-Mackler, Lynn; A 10-year prospective trial of a patient management algorithm and screening examination for highly active individuals with anterior cruciate ligament injury: Part 2, determinants of dynamic knee stability.; 2008; The American journal of sports medicine; 36; 1; 48-56;	Wrong outcomes
71	Hurd, Wendy J; Axe, Michael J; Snyder-Mackler, Lynn; A 10-year prospective trial of a patient management algorithm and screening examination for highly active individuals with anterior cruciate ligament injury: Part 1, outcomes.; 2008; The American journal of sports medicine; 36; 1; 40-7;	Wrong study design
72	Iliopoulos, Efthymios; Galanis, Nikiforos; Iosifidis, Michael; Zafeiridis, Andreas; Papadopoulos, Pericles; Potoupnis, Michael; Geladas, Nikolaos; Vrabas, Ioannis S; Kirkos, John; Anterior cruciate ligament deficiency reduces walking economy in "copers" and "non-copers".; 2017; Knee surgery, sports traumatology, arthroscopy : official journal of the ESSKA; 25; 5; 1403-1411; https://dx.doi.org/10.1007/s00167-015-3709-2	Wrong outcomes
73	Joseph, Clement; Pathak, Shirish S; Aravinda, M; Rajan, David; Is ACL reconstruction only for athletes? A study of the incidence of meniscal and cartilage injuries in an ACL-deficient athlete and non-athlete population: an Indian experience.; 2008; International orthopaedics; 32; 1; 57-61;	Wrong outcomes
74	Jungmann, Pia M; Baum, Thomas; Nevitt, Michael C; Nardo, Lorenzo; Gersing, Alexandra S; Lane, Nancy E; McCulloch, Charles E; Rummeny, Ernst J; Link, Thomas M; Degeneration in ACL Injured Knees with and without Reconstruction in Relation to Muscle Size and Fat Content-Data from the Osteoarthritis Initiative.; 2016; PloS one; 11; 12; e0166865; https://dx.doi.org/10.1371/journal.pone.0166865	Wrong comparator
75	Keays S.; Newcombe P.; Keays A. ; Return to Sport after Anterior Cruciate Ligament (ACL) Injury: Surgery versus No surgery. A long-term follow-up study; 2019; Journal of Science and Medicine in Sport; 22; Supplement 2; S59; http://dx.doi.org/10.1016/j.jsams.2019.08.255	Conference abstract
76	Keays, S. L.; Newcombe, P. A.; Bullock-Saxton, J. E.; Keays, A. C.; The development of long-term osteoarthritis following anterior cruciate ligament injury: reconstruction vs no reconstruction; 2023; Archives of Orthopaedic and Trauma Surgery; 143(6); ; 3201-3211; https://dx.doi.org/10.1007/s00402-022-04662-4	Wrong intervention
77	Kumar, M.A.N.; Nikose, S.; Saoji, K.; Palsodkar, P.; Outcome analysis of anterior cruciate ligament injury; 2019; International Journal of Pharmaceutical Research; 11; 4; 2080-2083; 10.31838/ijpr/2019.11.04.516	Conference abstract
78	Kvist, J.; Pettersson, M.; Knee-Related Quality Of Life 20 And 35 Years After An Anterior Cruciate Ligament Injury Treated Surgically Or Non-Surgically; 2023; Osteoarthritis and Cartilage; 31(Supplement 1); S171; https://dx.doi.org/10.1016/j.joca.2023.01.141	Conference abstract
79	Kvist, J.; Samuelsson Selin, A.; Faltstrom, A.; Gauffin, H.; Surgeries within two years after acute anterior cruciate ligament injury. results from the nacox-study; 2023; Osteoarthritis and Cartilage; 31(5); ; 692; https://dx.doi.org/10.1016/j.joca.2023.02.035	Conference abstract
80	Lambert, Christophe; Guenther, Daniel; SchÃ¼tz, Lisa-Marie; Kern, Niklas; Ritzmann, Ramona; Reinert, NoÃ©mie; Walz, Martin; Wafaisade, Arasch; Nagy, Kolos; Reuter, Sven; Psychological readiness is related to return to sport in judo injuries: a cross-sectional study; 2023; BMC Sports Science, Medicine & Rehabilitation; 15; 1; 1-Aug;	Wrong study design

81	Larose, G.; Leiter, J.; Peeler, J.; McRae, S.; Stranges, G.; Rollins, M.; Davidson, M.; MacDonald, P.; Quality of life during the wait for ruptured anterior cruciate ligament reconstruction: a randomized controlled trial; 2022; Canadian journal of surgery. Journal canadien de chirurgie; 65; 2; E269â€ E274; 10.1503/cjs.007820	Wrong comparator
82	Lee, J. H.; Han, S. B.; Park, J. H.; Choi, J. H.; Suh, D. K.; Jang, K. M.; Impaired neuromuscular control up to postoperative 1 year in operated and nonoperated knees after anterior cruciate ligament reconstruction; 2019; Medicine (United States); 98(15) (no pagination); https://dx.doi.org/10.1097/MD.0000000000015124	Wrong study design
83	Lee, Jin Hyuck; Han, Seung-Beom; Park, Jong-Hoon; Choi, Jae-Hyuk; Suh, Dae Keun; Jang, Ki-Mo; Impaired neuromuscular control up to postoperative 1 year in operated and nonoperated knees after anterior cruciate ligament reconstruction.; 2019; Medicine; 98; 15; e15124; https://dx.doi.org/10.1097/MD.0000000000015124	Wrong comparator
84	Lee, Song Joo; Ren, Yupeng; Chang, Alison H; Geiger, Francois; Zhang, Li-Qun; Effects of pivoting neuromuscular training on pivoting control and proprioception.; 2014; Medicine and science in sports and exercise; 46; 7; 1400-9; https://dx.doi.org/10.1249/MSS.0000000000000249	Wrong patient population
85	Lien-Iversen, Teodor; Morgan, Daniel Barklin; Jensen, Carsten; Risberg, May Arna; Engebretsen, Lars; Viberg, Bjarke; Does surgery reduce knee osteoarthritis, meniscal injury and subsequent complications compared with non-surgery after ACL rupture with at least 10 years follow-up? A systematic review and meta-analysis.; 2020; British journal of sports medicine; 54; 10; 592-598; https://dx.doi.org/10.1136/bjsports-2019-100765	Wrong study design
86	Lu, Y.; Jurgensmeier, K.; Till, S. E.; Reinholz, A.; Saris, D. B. F.; Camp, C. L.; Krych, A. J.; Early ACLR and Risk and Timing of Secondary Meniscal Injury Compared With Delayed ACLR or Nonoperative Treatment: A Time-to-Event Analysis Using Machine Learning; 2022; The American journal of sports medicine; 50(13); ; 3544-3556; https://dx.doi.org/10.1177/03635465221124258	Wrong study design
87	Madelaine, Anya; Fournier, Gaspard; Sappey-Marinier, Elliot; Madelaine, Thomas; Seil, Romain; Lefevre, Nicolas; Chotel, Franck; French Arthroscopic Society; Conservative management of anterior cruciate ligament injury in paediatric population: About 53 patients.; 2018; Orthopaedics & traumatology, surgery & research : OTSR; 104; 8S; S169-S173; https://dx.doi.org/10.1016/j.otsr.2018.09.001	Wrong outcomes
88	Marmura, H.; Tremblay, P. F.; Getgood, A. M. J.; Bryant, D. M.; The Knee Injury and Osteoarthritis Outcome Score Does Not Have Adequate Structural Validity for Use with Young, Active Patients with ACL Tears; 2022; Clinical Orthopaedics and Related Research; 480(7); ; 1342-1350; https://dx.doi.org/10.1097/CORR.0000000000002158	Wrong study design
89	McCarroll, J R; Rettig, A C; Shelbourne, K D; Anterior cruciate ligament injuries in the young athlete with open physes.; 1988; The American journal of sports medicine; 16; 1; 44-7;	Wrong intervention
90	Mei, Yu; Ao, Ying-fang; Wang, Jian-quan; Ma, Yong; Zhang, Xin; Wang, Jia-ning; Zhu, Jing-xian; Clinical characteristics of 4355 patients with anterior cruciate ligament injury.; 2013; Chinese medical journal; 126; 23; 4487-92	Wrong comparator
91	Messner, K.; Maletius, W.; Eighteen- to twenty-five-year follow-up after acute partial anterior cruciate ligament rupture; 1999; American Journal of Sports Medicine; 27; 4; 455-459; 10.1177/03635465990270040801	Wrong patient population
92	Meunier, A; Odensten, M; Good, L; Long-term results after primary repair or non-surgical treatment of anterior cruciate ligament rupture: a randomized study with a 15-year follow-up.; 2007; Scandinavian journal of medicine & science in sports; 17; 3; 230-7;	Wrong intervention
93	Mirza, F.; Mai, D.D.; Kirkley, A.; Fowler, P.J.; Amendola, A.; Management of injuries to the anterior cruciate ligament: results of a survey of orthopaedic surgeons in Canada.; 2000; Clinical Journal of Sport Medicine; 10; 2; 85-88;	Wrong outcomes

94	Moksnes, Havard; Engebretsen, Lars; Risberg, May Arna; Performance-based functional outcome for children 12 years or younger following anterior cruciate ligament injury: a two to nine-year follow-up study.; 2008; Knee surgery, sports traumatology, arthroscopy : official journal of the ESSKA; 16; 3; 214-23	Wrong comparator
95	Moon K.T. ; Early vs. Delayed treatment of anterior cruciate ligament tears; 2011; American Family Physician; 83; 7; 842-844	Wrong study design
96	Moon, H. S.; Choi, C. H.; Kim, S.; Yoo, J. H.; Jung, M.; Kwon, H. J.; Hong, Y. J.; Kim, S. H.; Outpatient-based diagnostic criteria for partial ACL injury: clinical outcomes of non-operative treatment and radiographic predictor; 2023; Archives of Orthopaedic and Trauma Surgery; 143(4); ; 2027-2036; https://dx.doi.org/10.1007/s00402-022-04467-5	Wrong intervention
97	Myklebust, Grethe; Holm, Inger; Maehlum, Sverre; Engebretsen, Lars; Bahr, Roald; Clinical, functional, and radiologic outcome in team handball players 6 to 11 years after anterior cruciate ligament injury: a follow-up study.; 2003; The American journal of sports medicine; 31; 6; 981-9;	Wrong comparator
98	NCT04408690,; Feasibility of a RCT That Compares Immediate Versus Optional Delayed Surgical Repair After ACL Injury; 2020; ; ; ; ;	Wrong study design
99	NCT04770233,; How to Best Treat Anterior Cruciate Ligament Injuries; 2021; ; ; ; ;	Wrong study design
100	Nisonson, B.; Goldberg, B.; Anterior cruciate ligament injuries. Conservative vs. surgical treatment. / Les lesions du ligament croise anterieur. Traitement conservateur contre traitement chirurgical.; 1991; Physician & Sportsmedicine; 19; 5; 82-89;	Wrong study design
101	Nl; Which patient with an anterior cruciate ligament rupture will need a surgical reconstruction?; 2020; http://www.who.int/trialsearch/Trial2.aspx?TrialID=NL8637	Conference abstract
102	NTR2746,; Cost-effectiveness of two treatment strategies of an anterior cruciate ligament rupture. A randomized clinical study; 2011; ; ; ; ;	Wrong study design
103	Odensten, M.; Hamberg, P.; Nordin, M.; Lysholm, J.; Gillquist, J.; Surgical or conservative treatment of the acutely torn anterior cruciate ligament. A randomized study with short-term follow-up observations.; 1985; Clinical Orthopaedics & Related Research; 198; 87-93;	Wrong intervention
104	Park, Yong-Geun; Ha, Chul-Won; Park, Yong-Beom; Na, Sang-Eun; Kim, Manyoung; Kim, Tae Seon; Chu, Yong Yeon; Is it worth to perform initial non-operative treatment for patients with acute ACL injury?: a prospective cohort prognostic study.; 2021; Knee surgery & related research; 33; 1; 11; https://dx.doi.org/10.1186/s43019-021-00094-3	Wrong study design
105	Pedersen M.; Grindem H.; Berg B.; Gunderson R.; Engebretsen L.; Axe M.J.; Snyder-Mackler L.; Risberg M.A. ; Low Rates of Radiographic Knee Osteoarthritis 5 Years After ACL Reconstruction or Rehabilitation Alone: The Delaware-Oslo ACL Cohort Study; 2021; Orthopaedic Journal of Sports Medicine; 9; 8; ; http://dx.doi.org/10.1177/23259671211027530	Wrong outcomes
106	Previ, L.; Monaco, E.; Carrozzo, A.; Fedeli, G.; Annibaldi, A.; Cantagalli, M. R.; Rossi, G.; Ferretti, A.; Spontaneous healing of a ruptured anterior cruciate ligament: a case series and literature review; 2023; Journal of Experimental Orthopaedics; 10(1) (no pagination); https://dx.doi.org/10.1186/s40634-022-00566-9	Wrong study design
107	Rambaud A.J.; Neri T.; Edouard P. ; Reconstruction, rehabilitation and return-to-sport continuum after anterior cruciate ligament injury (ACLR3-continuum): Call for optimized programs; 2022; Annals of Physical and Rehabilitation Medicine; 65; 4; 101470; http://dx.doi.org/10.1016/j.rehab.2020.101470	Wrong study design

108	Reijman, Max; Eggerding, Vincent; van Es, Eline; van Arkel, Ewoud; van den Brand, Igor; van Linge, Joost; Zijl, Jacco; Waarsing, Erwin; Bierma-Zeinstra, Sita; Meuffels, Duncan; Early surgical reconstruction versus rehabilitation with elective delayed reconstruction for patients with anterior cruciate ligament rupture: COMPARE randomised controlled trial.; 2021; BMJ (Clinical research ed.); 372; 8900488, bmj, 101090866; n375; https://dx.doi.org/10.1136/bmj.n375	Wrong intervention
109	Roos, Paulien E; Button, Kate; Sparkes, Valerie; van Deursen, Robert W M; Altered biomechanical strategies and medio-lateral control of the knee represent incomplete recovery of individuals with injury during single leg hop.; 2014; Journal of biomechanics; 47; 3; 675-80; https://dx.doi.org/10.1016/j.jbiomech.2013.11.046	Wrong outcomes
110	Sailhan F.; Ribinik P. ; Conservative versus operative treatment for anterior cruciate ligament tear: Results and risk factors for osteoarthritis; 2015; Annals of Physical and Rehabilitation Medicine; 58; SUPPL. 1; e67-e68; http://dx.doi.org/10.1016/j.rehab.2015.07.155	Wrong study design
111	Sanchez Romero E.A.; Lim T.; Perez J.L.A.; Castaldo M.; Lozano P.M.; Villafane J.H. ; Identifying clinical and MRI characteristics associated with quality of life in patients with anterior cruciate ligament injury: Prognostic factors for long-term; 2021; International Journal of Environmental Research and Public Health; 18; 23; 12845; https://dx.doi.org/10.3390/ijerph182312845	Wrong study design
112	Sandberg R; Balkfors B; Nilsson B; Westlin N; Operative versus non-operative treatment of recent injuries to the ligaments of the knee. A prospective randomized study; 1987; Journal of Bone and Joint Surgery -- American Volume 1987 Oct; 69(8):1120-1126	Wrong intervention
113	Scavenius, M; Bak, K; Hansen, S; Norring, K; Jensen, K H; Jorgensen, U; Isolated total ruptures of the anterior cruciate ligament--a clinical study with long-term follow-up of 7 years.; 1999; Scandinavian journal of medicine & science in sports; 9; 2; 114-9;	Wrong study design
114	Schabus R. ; The management of the isolated injury of the anterior cruciate ligament - Long-term results; 1996; Acta Chirurgica Austriaca; 28; SUPPL. 120; 130-133	Wrong intervention
115	Secrist, Eric S; Frederick, Robert W; Tjoumakaris, Fotios P; Stache, Stephen A; Hammoud, Sommer; Freedman, Kevin B; A Comparison of Operative and Nonoperative Treatment of Anterior Cruciate Ligament Injuries.; 2016; JBJS reviews; 4; 11; ; https://dx.doi.org/10.2106/JBJS.RVW.15.00115	Wrong study design
116	Seitz, H; Chrysopoulos, A; Egkher, E; Mousavi, M; [Long-term results of replacement of the anterior cruciate ligament in comparison with conservative therapy].; 1994; Der Chirurg; Zeitschrift fur alle Gebiete der operativen Medizen; 65; 11; 992-8;	Study not in English
117	Seng, Khemarin; Appleby, David; Lubowitz, James H; Operative versus nonoperative treatment of anterior cruciate ligament rupture in patients aged 40 years or older: an expected-value decision analysis.; 2008; Arthroscopy : the journal of arthroscopic & related surgery : official publication of the Arthroscopy Association of North America and the International Arthroscopy Association; 24; 8; 914-20; https://dx.doi.org/10.1016/j.arthro.2008.01.021	Wrong study design
118	Shelton, W R; Barrett, G R; Dukes, A; Early season anterior cruciate ligament tears. A treatment dilemma.; 1997; The American journal of sports medicine; 25; 5; 656-8;	Wrong study design
119	Smale, Kenneth B.; Flaxman, Teresa E.; Alkjaer, Tine; Simonsen, Erik B.; Krogsgaard, Michael R.; Benoit, Daniel L.; Anterior cruciate ligament reconstruction improves subjective ability but not neuromuscular biomechanics during dynamic tasks.; 2019; Knee Surgery, Sports Traumatology, Arthroscopy; 27; 2; 636-645	Wrong study design
120	Snyder-Macker, L.; Fitzgerald, G.K.; Axe, M.; Lear, L.; ACL repair in athletes: who can play now and get fixed later? (Abstract); 1998; Medscape Orthopaedics & Sports Medicine; 2; 2; ;	Not published in a journal

121	Stockton D.J.; Schmidt A.M.; Yung A.; Desrochers J.; Zhang H.; Masri B.A.; Wilson D.R. ; Tibiofemoral contact and alignment in patients with anterior cruciate ligament rupture treated nonoperatively versus reconstruction: An upright, open MRI study; 2021; Bone and Joint Journal; 103 B; 9; 1505-1513; https://dx.doi.org/10.1302/0301-620X.103B9.BJJ-2020-1955.R1	Wrong outcomes
122	Stockton, D. J.; Schmidt, A. M.; Yung, A.; Desrochers, J.; Zhang, H.; Masri, B. A.; Wilson, D. R.; Tibiofemoral contact and alignment in patients with anterior cruciate ligament rupture treated nonoperatively versus reconstruction: An upright, open MRI study; 2021; Bone and Joint Journal; 103 B(9); 1505-1513; https://dx.doi.org/10.1302/0301-620X.103B9.BJJ-2020-1955.R1	Wrong intervention
123	Strehl, Alexander; Eggli, Stefan; The value of conservative treatment in ruptures of the anterior cruciate ligament (ACL).; 2007; The Journal of trauma; 62; 5; 1159-62	Wrong intervention
124	Swirtun, L R; Renstrom, P; Factors affecting outcome after anterior cruciate ligament injury: a prospective study with a six-year follow-up.; 2008; Scandinavian journal of medicine & science in sports; 18; 3; 318-24	Wrong intervention
125	Tengman E.; Persson M.; Hager C. ; Knee muscle strength and jump capacity in subjects with ACL-injury about 24 years after injury; 2011; Physiotherapy (United Kingdom); 97; SUPPL. 1; eS1222-eS1223; http://dx.doi.org/10.1016/j.physio.2011.04.002	Conference abstract
126	Thoma L.; Johnson J.; White D.; Risberg M.A.; Snyder-Mackler L. ; Factors associated with overweight or obesity in athletes 5 years after anterior cruciate ligament injury; 2018; Arthritis and Rheumatology; 70; Supplement 9; 2186-2187; http://dx.doi.org/10.1002/art.40700	Conference abstract
127	Thoma L.M.; Snyder-Mackler L.; Risberg M.; White D.K. ; Trajectories of weight gain in young adults following anterior cruciate ligament rupture: the delaware-Oslo ACL cohort study; 2019; Osteoarthritis and Cartilage; 27; Supplement 1; S274-S275; http://dx.doi.org/10.1016/j.joca.2019.02.653	Conference abstract
128	Thoma, Louise M; Grindem, Hege; Logerstedt, David; Axe, Michael; Engebretsen, Lars; Risberg, May Arna; Snyder-Mackler, Lynn; Coper Classification Early After Anterior Cruciate Ligament Rupture Changes With Progressive Neuromuscular and Strength Training and Is Associated With 2-Year Success: The Delaware-Oslo ACL Cohort Study.; 2019; The American journal of sports medicine; 47; 4; 807-814; https://dx.doi.org/10.1177/0363546519825500	Wrong outcomes
129	Thomee, R; Walden, M; Hagglund, M; Return to sports after anterior cruciate ligament injury: neither surgery nor rehabilitation alone guarantees success--it is much more complicated.; 2015; British journal of sports medicine; 49; 22; 1422; https://dx.doi.org/10.1136/bjsports-2015-094793	Wrong study design
130	Thorstensson, CA; Lohmander, LS; Frobell, RB; Roos, EM; Gooberman-Hill, R; Choosing surgery: patients' preferences within a trial of treatments for anterior cruciate ligament injury. A qualitative study; 2009; ; 10; ; 100; 10.1186/1471-2474-10-100	Wrong study design
131	Truong L.K.; Mosewich A.D.; Miciak M.; Pajkic A.; Le C.Y.; Li L.C.; Whittaker J.L. ; Balance, reframe, overcome. attitudes, priorities and perceptions toward physical activity and exercise-therapy in youth 12-24 months after a sport-related anterior cruciate ligament injury; 2021; Osteoarthritis and Cartilage; 29; Supplement 1; S24-S25; http://dx.doi.org/10.1016/j.joca.2021.02.048	Wrong study design
132	Ucay O.; Gleizes Cervera S.; Renault A.; Gasq D. ; Barriers to the return to sport after anterior cruciate ligament tear in operative vs. Conservative patients; 2015; Annals of Physical and Rehabilitation Medicine; 58; SUPPL. 1; e68; http://dx.doi.org/10.1016/j.rehab.2015.07.156	Conference abstract
133	Ucay O.; Renault A.; Cervera S.G.; Gasq D. ; How to evaluate precisely return to sport after anterior cruciate ligament tear with operative or conservative treatment on patients with moderate sport level?; 2016; Annals of Physical and Rehabilitation Medicine; 59; Supplement; e19; http://dx.doi.org/10.1016/j.rehab.2016.07.046	Conference abstract

134	Urhausen, A. P.; Pedersen, M.; Grindem, H.; Ito, N.; Arhos, E.; Smith, A.; Silbernagel, K.; Axe, M.; Engebretsen, L.; Snyder-Mackler, L.; Risberg, M.; Good 10-Year Outcomes Following The Treatment Algorithm Of The Delaware-Oslo ACL Cohort: Knee Osteoarthritis, Symptoms And Function; 2023; Osteoarthritis and Cartilage; 31(Supplement 1); S48-S49; https://dx.doi.org/10.1016/j.joca.2023.01.541	Conference abstract
135	Van Der Graaff, S.; Meuffels, D. E.; Bierma-Zeinstra, S.; Van Es, E.; Verhaar, J.; Eggerding, V.; Reijman, M.; Why, When and Who Fails Non-Operative Treatment of Anterior Cruciate Ligament Injury - an Exploratory Analysis of the Compare Trial; 2021; Journal of ISAKOS; 6(6); ; 478-479; https://dx.doi.org/10.1136/jisakos-2021-congress.206	Conference abstract
136	van der Graaff, Sabine J A; Meuffels, Duncan E; Bierma-Zeinstra, Sita M A; van Es, Eline M; Verhaar, Jan A N; Eggerding, Vincent; Reijman, Max; Why, When, and in Which Patients Nonoperative Treatment of Anterior Cruciate Ligament Injury Fails: An Exploratory Analysis of the COMPARE Trial.; 2022; The American journal of sports medicine; 50; 3; 645-651; https://dx.doi.org/10.1177/03635465211068532	Wrong study design
137	van der List, Jelle P; Hagemans, Frans J A; Hofstee, Dirk Jan; Jonkers, Freerk J; The Role of Patient Characteristics in the Success of Nonoperative Treatment of Anterior Cruciate Ligament Injuries.; 2020; The American journal of sports medicine; 48; 7; 1657-1664; https://dx.doi.org/10.1177/0363546520917386	Wrong outcomes
138	van Meer, Belle L; Oei, Edwin H G; Meuffels, Duncan E; van Arkel, Ewoud R A; Verhaar, Jan A N; Bierma-Zeinstra, Sita M A; Reijman, Max; Degenerative Changes in the Knee 2 Years After Anterior Cruciate Ligament Rupture and Related Risk Factors: A Prospective Observational Follow-up Study.; 2016; The American journal of sports medicine; 44; 6; 1524-33; https://dx.doi.org/10.1177/0363546516631936	Wrong outcomes
139	Vasile, Manole; LĂƒcrĂƒmioara, Manole; Marius, Manole; POSSIBILITIES OF RECOVERY BY MEANS OF PHYSIOTHERAPY AFTER ANTERIOR CRUCIATE LIGAMENT PLASTY (KENETH-JONES) AT HANDBALL PLAYERS.; 2011; Gymnasium: Journal of Physical Education & Sports; 12; 2; Jul-14;	Wrong study design
140	Wasmaier, J.; Eid, K.; Kubik-Huch, R.; Bieg, C.; Pfirrmann, C.; Grehn, H.; Proximal Anterior Cruciate Ligament Tears: The Healing Response Technique versus Conservative Treatment; 2012; Journal of Knee Surgery; 26; 4; 263-272; 10.1055/s-0032-1329720	Wrong intervention
141	Webster, Kate E; Feller, Julian A; A research update on the state of play for return to sport after anterior cruciate ligament reconstruction.; 2019; Journal of orthopaedics and traumatology : official journal of the Italian Society of Orthopaedics and Traumatology; 20; 1; 10; https://dx.doi.org/10.1186/s10195-018-0516-9	Wrong study design
142	Wellsandt, Elizabeth; Axe, Michael J; Snyder-Mackler, Lynn; Poor Performance on Single-Legged Hop Tests Associated With Development of Posttraumatic Knee Osteoarthritis After Anterior Cruciate Ligament Injury.; 2018; Orthopaedic journal of sports medicine; 6; 11; 2.33E+15; https://dx.doi.org/10.1177/2325967118810775	Wrong study design
143	Wellsandt, Elizabeth; Failla, Matthew J; Axe, Michael J; Snyder-Mackler, Lynn; Does Anterior Cruciate Ligament Reconstruction Improve Functional and Radiographic Outcomes Over Nonoperative Management 5 Years After Injury?.; 2018; The American journal of sports medicine; 46; 9; 2103-2112; https://dx.doi.org/10.1177/0363546518782698	Wrong intervention
144	Winterstein, Andrew P.; McGuine, Timothy A.; Carr, Kathleen E.; Hetzel, Scott; Changes in Self-reported Physical Activity Following Knee Injury in Active Females.; 2013; Athletic Training & Sports Health Care: The Journal for the Practicing Clinician; 5; 3; 106-114; 10.3928/19425864-20130412-01	Wrong patient population
145	Wittenberg, R H; Oxfort, H U; Plafki, C; A comparison of conservative and delayed surgical treatment of anterior cruciate ligament ruptures. A matched pair analysis.; 1998; International orthopaedics; 22; 3; 145-8;	Wrong comparator
146	Zatterstrom R; Friden T; Lindstrand A; Moritz U; Rehabilitation following acute anterior cruciate ligament injuries -- a 12-month follow-up of a randomized clinical trial; 2000; Scandinavian Journal of Medicine & Science in Sports 2000 Jun; 10(3):156-163	Wrong intervention

147	Zatterstrom, R.M.; The injured anterior cruciate ligament and neuromuscular rehabilitation.; 1999	Study not in English
148	Zysk, S P; Refior, H J; Operative or conservative treatment of the acutely torn anterior cruciate ligament in middle-aged patients. A follow-up study of 133 patients between the ages of 40 and 59 years.; 2000; Archives of orthopaedic and trauma surgery; 120; 1-Feb; 59-64	Wrong intervention
