# Supplementary material for: No Difference in Return-to-Sport Rate or Activity Level in People with Anterior Cruciate Ligament (ACL) Injury Managed with ACL Reconstruction or Rehabilitation Alone: A Systematic Review and Meta-Analysis
Source: Sports Med. 2025 Jul 2;55(9):2191–205. doi: 10.1007/s40279-025-02268-5 (PMC12476414; doi:10.1007/s40279-025-02268-5)
Supplement: Supplementary file 2 — Supplementary file2 (PDF 135 KB) [file 40279_2025_2268_MOESM2_ESM.pdf]

Supplementary Appendix 2

Study characteristics and results

Study name	Design	Study sample	Treatment allocation/selection	Early/initial ACLR group	Rehab alone group	RTS recommendations	Rehabilitation description	Return to sport and activity level measure/s and results	Follow-up period
Fink et al., 2001[35]	Retrospective cohort study	n=113	Surgery generally recommended for those under 40 years	n=72 (n=46 attended final follow-up)<br><br>20% female<br><br>Mean age 34 years (SD 8)<br><br>Surgery performed within 6 months of injury, mean 3.3 months (SD 2.7)	n=41 (n=25 attended final follow-up)<br><br>28% female<br><br>Mean age 32 years (SD 10)	None reported	Postop immobilization in 20° knee flexion for six weeks with partial weightbearing, followed by a structured physical therapy protocol for an average time of 7.0 weeks (SD 3.4).<br><br>Rehab alone group: Home exercise program focussed on hamstring strengthening, cycling and swimming. ROM if required. Reviewed by physical therapist up to 6 months.	Innsbruck Knee Sports Rating Scale (3 sporting categories based on demands on knee stability) assessed sport participation overall: ACLR = 26% decrease in pre-injury activity levels, Rehab alone = 54% decrease in pre-injury activity levels.	10-13 years after injury
Fithian et al., 2005[32]	Prospective cohort	n=209<br><br>52% female<br><br>Mean age 39 (range 16-69) years	Patients were assigned to 1 of 3 groups (high, moderate, or low risk) based on initial knee stability testing and their preinjury levels of sports participation. Early ACLR defined as within 3 months of ACL injury, was recommended to those patients in the high-risk category, whereas conservative treatment was recommended to those in the low-risk group. The moderate-risk group, received either	Early ACLR (<3 months since injury) n=63	n=113	In the rehab alone group, patients were encouraged to avoid any competitive situations (including one-on-one drills and scrimmages, in addition to games) for 3 months. They were permitted to participate in all sports at 3 months unless symptomatic with IKDC level I and II activities	Weight-bear as tolerated. Started immediately on a program of non-impact closed-chain strengthening and ROM exercises. Allowed to start jogging and performing sport-specific drills between 6 and 12 weeks after injury.	TAS score: ACLR median 6, Rehab alone median 3<br><br>Return to sport rates: ACLR 52%, Rehab alone 52% returned to preinjury or higher levels of activity - assessed as same or higher TAS score at follow-up compared to pre-injury	Mean 6.6 years (range 3-10 years)

			treatment based on the surgeon's preference						
Frobell et al., 2010[8]	RCT	n=119<br><br>26% female<br><br>Mean age 26 years	Randomisation to ACLR or optional delayed ACLR)	Early ACLR, within 10 weeks of injury, n=60	n=36 (offered delayed ACLR but did not have surgery)	Assume no different between groups	Goal-based progressive exercise-based rehabilitation supervised by physiotherapist to improve ROM, muscle function and functional performance. Progression through four levels determined by meeting pre-specified targets.	TAS score: ACLR median 6.5 (IRQ 3-8); Rehab alone median 5 (IRQ 4-8)<br><br>Return to previous activity (%): ACLR: 27 (44%); Rehab alone 14 (39%) assessed as same or higher TAS score at follow-up compared to pre-injury	ACLR mean 24.6 months (SD 24.4-24.7), Rehab alone mean 24.9 months (SD 24.5-25.2)
Frobell et al., 2013[9]	RCT (extended follow-up from Frobell et al., 2010)	n=118<br><br>26% female<br><br>Mean age 26 years	a/a	n=59	n=29 (offered delayed ACLR but did not have surgery)	a/a	a/a	TAS score: ACLR median 4 (IRQ 2-7), Rehab alone median 4 (IRQ 2-6.5)<br><br>Return to pre-injury activity level (%), assessed as same or higher TAS score at follow-up compared to pre-injury; ACLR: 13 (23%), Rehab alone 6 (21%)	ACLR mean 60 months (95%CI 59-61),<br>Rehab alone: 58 months (95%CI 55-61)
Grindem et al 2012[34]<br><br>Note: Participants were also included in either Moksnes 2008/9 or Pedersen 2021	Retrospective pair-matched cohort study	n=138<br><br>46% female<br><br>Mean age: 28 (SD 7) years<br><br>ACL rupture in previous 6 months<br><br>Participated in pivoting sports at least twice/week pre-injury	Retrospective pair-matched on specific preinjury sport, sex, and age.<br><br>Non-operative or operative treatment determined by orthopaedic surgeon after initial rehab period based on a wish to return to level I sports, dynamic instability, young age (but skeletally mature), and patient's preference for surgery.	n=69	n=69	RTS criteria were at least 90% hamstring and quadriceps strength, and LSI ≥90% on 4 single-leg hop tests. ACLR patients recommended not to return to level II sports earlier than 6 months postoperatively, and to level I sports no earlier than 9 months postop. Rehab alone patients could return to level II or lower sports as soon as the return to sports criteria are met; recommended to refrain from returning to level I sports without ACLR.	Rehab alone patients undergo 3 to 4 months of rehabilitation after initial impairments have resolved. Discharged when they meet specific criteria for quadriceps and hamstring strength (LSI ≥90% on 4 single-legged hop tests.<br><br>Note: All participants level I sports pre-injury but only Rehab alone group told not to return to level I sports.	Return to sport rates: (defined as return to same or higher level of physical activity)<br><br>Overall return to sport: ACLR 68% (47/69), Rehab alone 68% (47/69)<br><br>Preinjury sports activity was defined as the patient's self-reported main sports activity before injury. The patient was registered as having returned to sport if his or her sports activity at follow-up matched the preinjury main sports activity. Patients who returned to sports other	1 year

								than their preinjury main sports activity were thus classified as not having returned to sport.<br><br>Return to level I (jumping/cutting/pivoting) sport [b]: ACLR 62% (26/42), Rehab alone 55% (23/42)<br><br>Return to level II (lateral movements, less pivoting) sport [b]: ACLR 78% (21/27), Rehab alone 89% (24/27)	
Grindem 2014[14]	Prospective cohort	n=143<br><br>56% female<br><br>Age 13-60 years<br><br>Participated in level-I or II sports twice a week or more	Patients chose surgical or non-surgical treatment after initial symptom management period and the main reason for their choice was recorded.	n=100<br><br>56% female	n=43<br><br>56% female	All patients advised not to return to level-I or II sports until LSI >90% for hamstring and quadriceps strength and for four hop tests.<br><br>ACLR patients recommended to avoid level-II sports for >6 months and avoid level-I sports for t>9 months. Rehab alone patients advised not to participate in any level-I sports forever.<br><br>Rehab focused on strength and neuromuscular training and plyometrics for 6-12 months.	a/a<br>Rehab typically lasted 2-3 months	Monthly level-I (jumping/cutting/pivoting) sports participation: Crude odds ratio 2.78 (95%CI 1.40, 5.52)<br><br>Propensity-Score-adjusted* odds ratio 1.3 (0.61-2.78)<br><br>Monthly level-II (lateral movements, less pivoting) sports participation: Crude odds ratio 0.65 (95%CI (0.37-1.14)<br><br>Propensity-Score-adjusted* odds ratio 0.88 (0.47-1.34)<br><br>(OR >1 indicates more ACLR patients participating)<br><br>*adjusted for preinjury participation in level-I sports, preinjury participation in level-II sports, age, sex, BMI, and concomitant injuries	2 years
Keays et al., 2022[25]	Data from two case series<br>1. ACLR<br>2. Rehab alone	n=93 of 101	Rehab alone patients were recruited from surgeons' records who had originally elected not	n=51 of 56<br><br>30% female	n=42 of 45	Rehab alone patients encouraged not to return to competitive sports, this advice was	Physiotherapy focusing on full ROM, eccentric quadriceps control,	TAS score: ACLR mean 6.9 (range 2-9); Rehab alone mean 5.5 (range 2-9).	ACLR: median 9 years after injury (IQR 7-

			to have surgery due to work demands, lower physical demand, older age, surgeon's advice or because they were functionally stable (copers).<br><br>Different exclusion criteria applied to each group.	Mean age 33 (range 24-47) years<br><br>All patients had preoperative rehabilitation, median time from injury to surgery was 2.5 years (IQR 1-4)	33% female<br><br>Mean age 38 (range 23-49) years	not given to ACLR patients. ACLR patients did progressive running, agility training and sport-specific training before undergoing return to sport testing. RTS criteria for ACLR only were: full ROM, 90% quadriceps and hamstring muscle strength index, 90% hop index for single and triple hop for distance, and specific times for 3 agility tests	muscle strength, and dynamic stability. Similar program to ACLR group but attended for less time.	Return to any sport: ACLR 96%, Rehab alone 93%.<br><br>Return to same sport or higher level of sport: ACLR 65%, Rehab alone 26%.<br><br>Pre-injury and current sporting level was assessed on a 6-point pivoting scale (where 0=no sport, 5=vigorous pivoting team sports at a competitive level)	11), Rehab alone: median 11 years after injury (IQR 7-14)
Kessler et al., 2008[24]	Retrospective cohort study	n=109<br><br>38% female<br><br>Mean age: 31 (range 13-54) years	Treatment based on consensus between the surgeon and the patient independent of the sports activity level (based on fear of operative complications and occupation related factors).<br><br>Patients who underwent a 'revision operation' within 11 years were excluded from the study.	n=60	n=49	All patients advised to participate in sports activities regardless of group.<br><br>The criteria for return to sports were no pain, no swelling during or after loading, and no sense of instability.	Both groups followed the same rehabilitation program.<br>Wearing an ACL brace for 6 weeks, training of hamstrings and quadriceps, proprioception training, no flexion under load greater than 60° for 6 weeks, return to sports activities after 3 months, and return to contact/pivoting sports after 9 months.	TAS score: ACLR mean 5.3 (range 2.0-10), Rehab alone mean 4.9 (range 2.0-10)<br><br>TAS score as a percentage: ACLR 13% 9-10, 7% 7-8, 27% 5-6, 47% 3-4, 6% 1-2, Rehab alone 12% 9-10, 6% 7-8, 43% 5-6, 35% 3-4, 4% 1-2.	Mean 11.1 years (range 7.5-16.3)
Kovalak et al 2018[15]	Retrospective cohort study.<br><br>Match-paired groups were formed based on sex, age, BMI, and activities.	n=82<br><br>0% female<br><br>Mean age: 32 years<br><br>TAS score 3-7, only recreational sporting activities, and no	Group allocation based on clinical and patient-related factors including fear of operative complications and occupation related issues. Patients with little side-to-side laxity (1þ) and recreational sporting activity levels were counselled on both treatment strategies. Treatment was	n=43 (ACLR 6-8 weeks post injury)	n=39	All patients were advised to avoid contact sports, irrespective of treatment strategy. Jogging was allowed after 12 weeks, and pivot sports allowed after 6 months in both groups.	Post op rehabilitation: Knee brace for 2 weeks, strength training commenced 1 week post-op, balance training, cycling and coordination exercises commenced after 3-4 weeks, resisted knee flexion/extension after 6 weeks. Mean rehab duration 7 months (range 6–8).	Time to return to sport: ACLR mean 12 months (10-16), Rehab alone mean 13 months (10-17)<br><br>Method for assessing return to sport was not reported	Minimum 5 years, ACLR 8.25 years (SD 1.82), Rehab alone 8.10 years (SD 2.43)

		concomitant injuries	determined by the surgeon and patient independent of athletic activity.				Rehab alone: 3 weeks in a knee brace, then supervised neuromuscular training to improve functional stability (specific activities, level of training, and progression schedule guided by neuromuscular performance). Mean rehab duration 7.2 months (range 6-9). Jogging allowed after 12 weeks, and pivot sports allowed after 6 months in both groups. Contact sports were to be avoided.		
Meuffels et al., 2009[31]	Matched pairs from 2 separate cohort studies	n=50<br><br>24% female<br><br>High level athletes, pre-injury TAS score median 9 (range 6-10).	Matched with respect to age, gender and TAS score before injury.<br><br>All patients initially had physiotherapist-led rehabilitation programme. After 3 months assessed for knee instability (persistent giving way) and offered a non-pivoting activity-lifestyle or ACLR	n=25<br><br>Mean age 38 (SD 6) years	n=25<br><br>Mean age 38 (SD 7) years	Rehab alone: Non-pivoting activity-lifestyle.<br>ACLR: could return to pivoting activities, sports return was allowed after 6 months.	Initially all patients swelling reduction, ROM exercises, hamstring and quadriceps strengthening program.<br><br>Rehab alone: Advised to do supervised rehab for at least 3 months.	Highest TAS score returned to post ACL injury: ACLR median 8 (range 3-10), Rehab alone median 7 (range 4-10)<br><br>TAS score at 10 years: ACLR median 6 (range 3-9), Rehab alone median 5 (range 1-9)	10 years<br><br>ACLR 10.3 years (9.7-11.4); Rehab alone 12 years (11.0-13.0)
van Yperen et al., 2018[30]	a/a	a/a	a/a	n=25<br><br>Mean age: 46 (SD 6) years	n=25<br><br>Mean age: 49 (SD 7) years	a/a	a/a	TAS score: ACLR median 5 (IRQ 3-6), Rehab alone median 4 (IRQ 4-6)	At 20 year assessment: ACLR median 21.2 years (IQR 20.0-22.8), Rehab alone median 24.1 years (IQR 22.6-27.0)

Mihelic et al., 2011[36]	Retrospective cohort	n=54<br><br>19% female	All patients advised ACLR or to modify activities, treatment group chosen by patient.	n=33<br><br>Mean age 25 years<br><br>Baseline TAS score mean 6 (range 6–9)<br><br>Surgery mean 19 months (range 3-24) post injury	n=18<br><br>Mean age 26 years<br><br>Baseline TAS score mean 5 (range 2–9)	Rehab alone patients advised to modify their sports and work activities.	Post op rehabilitation: Treated with paster case for 2 weeks and then rehab. Rehab alone: Treated with plaster cast for 3 weeks after initial arthroscopy ± meniscectomy, then 2 months rehab aiming to regain full ROM and strengthen quadriceps.	TAS score: ACLR mean 5 (range 2-9), Rehab alone mean 4 (range 2-6)	17-20 years since ACL rupture
Moksnes et al 2008[28]	Prospective cohort	n=125 (n=102, 82% completed 1 year assessment)<br><br>45% female<br><br>mean age: 27 (SD 9) years<br><br>People taking part in level I and II sports, aged 14-60 years	Activity level, type of activities, the number of giving-way episodes, age, the subject's own preferences, and the results from the screening examination were all considered when deciding whether to perform surgery or not.	n=50<br><br>52% female;<br><br>Time from injury to ACLR mean 184 (SD 91) days	n=52<br><br>44% female	None reported	All patients were enrolled in rehabilitation and encouraged to consult a physical therapist once/week for program progression. Home exercises to perform daily. Rehabilitation and preoperative physical therapy free for the first 6 months after ACL injury in Norway, and postoperative rehabilitation is similarly free for 6 months after ACLR. Physical therapist decides how many sessions are necessary. Rehab focused on muscle strengthening, agility drills, and neuromuscular training.	Return to pre-injury activity level: ACLR 70%, Rehab alone 65%.<br><br>Used an Activity Level Classification (4 levels of activity; level 1 = cutting/pivoting/jumping sports) to determine if they had returned to pre-injury level	1 year after study inclusion for rehab patients, 1 year after surgery for ACLR patients.
Moksnes et al 2009[29]	a/a	a/a	a/a	a/a	a/a	a/a	a/a	Return to pre-injury activity level at 12 months: ACLR 35/50 (70%), Rehab alone 36/52 (69%);<br><br>Used an Activity Level Classification (4 levels of activity) to determine if they	a/a

								had returned to pre-injury level<br><br>Activity level at follow-up (levels from 1-4 [c]): ACLR median 1 (95%CI 1-2), Rehab alone median 2 (95%CI 1-2)	
Pedersen et al 2021[33]	Prospective cohort	n=222 (from 276, 20% lost to follow-up)<br><br>n=142 from Oslo and n=134 from Delaware<br><br>Inclusion criteria: Aged 13-60 years; Preinjury participation in level-I or II sports ≥2 times/week.<br>- resolution of acute impairments (no or minimal pain or effusion during or after plyometric activities)<br>- within 3 months of injury [Oslo] or within 7 months of injury [Delaware]	After 5 weeks of prehabilitation and impairment resolution, patients received information about treatment alternatives. Treatment decision made with advice from surgeons and physical therapists. Patients experiencing dynamic knee instability after preoperative rehabilitation and those who intended to return to level-I sports were advised surgery. Delayed ACLR was indicated for persistent dynamic knee instability or if they changed their minds about the treatment choice that they had made.<br><br>Fewer people in the rehab alone group were participating in level I sports preinjury: ACLR 129 (77%), delayed ACLR 25 (83%), Rehab alone 30 (46%).	n=135 (19% lost to follow-up)<br><br>46% female<br><br>Age: 25 (SD 9) years	n=64 (2% lost to follow-up)<br><br>55% female<br><br>Age: 32 (SD 11) years	None reported.	After impairment resolution (mean 59 days after injury) all patients participated in a 5-week (10-session) rehabilitation program consisting of progressive neuromuscular and strength training exercises.<br><br>Postop rehabilitation: Phase 1 - Acute postoperative phase addressed swelling, range of motion, and atrophy. Phase 2 - Rehabilitation phase aimed to achieve a muscle strength and hop performance LSI >80%. Phase 3 - Return-to-sport phase included gradually increased participation in sports specific training; milestones were a strength and hop LSI of >90%.<br><br>Rehab alone: Continued progressive rehabilitation for 3 to 4 months with the same phases.	Return to preinjury sports participation: ACLR 64/135 (47%), Rehab alone 30/64 (47%), p=0.159<br><br>Current sports participation was assessed with the 'Sports Activity Classification' which used the following item: "What sports or exercise are you participating in now? (graded the most knee-demanding sport from I (jumping/cutting/pivoting) to 4)<br><br>Marx activity rating scale [d]: ACLR mean 8 (SD 4), Rehab alone mean 7 (SD 4), p=0.314.	5 years (ACLR 5.5, SD 0.5; Rehab alone 5.4, SD 0.5).
Streich et al 2011[37]	Retrospective matched (age,	n=80	Decision for ACLR was based on clinical and	n=40	n=40	Rehab alone patients were instructed to avoid	In all patients a diagnostic arthroscopy	TAS score: ACLR, mean 4.7 (SD 1.8, range 3-7), Rehab	15 years (range 14-16)

	gender, BMI, concomitant injuries) pairs case series	30% female	patient-related factors. All patients informed about both treatment options. ACLR was chosen if the patient participated in sports at a professional level (n=14), took part in sport activities including pivoting, cutting or sidestepping and were not willing to decrease their activity level (n=29), or an explicit wish to have a primary ACLR (n=24).	Mean age 26 (SD 6) years<br><br>Mean interval between injury and treatment 7.3 months (SD 3.2, range 2-29)	Mean age 24 (SD 7) years	high demand pivot shift activities such as downhill skiing, soccer and comparable sports.	and the ACL was debrided.<br><br>Postop rehabilitation: Immobilised in plaster cast for 14 days, ROM and isometric muscle exercises started and gradually progressed. Knee flexion of more than 90° and full weight bearing was allowed after 2 months. Indoor cycling and swimming were permitted after 8 weeks, running after 16 weeks, high-demand pivoting after 9 months.<br><br>Rehab consisted of neuromuscular based program supervised by physiotherapist to improve joint mobility and stability.	alone, mean 5.1 (SD 1.9, range 3-6)	
Tengman et al 2014[38]	Cross-sectional retrospective cohort study	n=70 (plus 33 non-injured subjects in control group)<br><br>36% female<br><br>Mean age 47 (SD 5) years	One hospital had a strategy that ACLR should be restricted only to those who would gain clear benefit from surgery and not merely be indicated based on severity of injury or activity level demands. This cohort was treated only with physiotherapy (Rehab alone).<br><br>In the other hospital, the indication for surgery was less restrictive and in this cohort all patients had surgery followed by post-operative physiotherapy (ACLR).	n=33<br><br>36% female<br><br>Mean age 46 (SD 5) years<br><br>3 months of pre-op rehab, ACLR, post-op rehab.<br><br>ACLR at mean 3.6 years (SD 2.3) after injury.	n=37<br><br>38% female<br><br>Mean age 48 (SD 6) years	Rehab alone group advice involved activity modification where some sports, such as soccer, floorball, and wrestling, were discouraged.<br><br>ACLR advice involved full return to sports activities from 22 weeks after surgery.	All patients were initially treated with physiotherapy for at least 3 months.<br><br>Rehab alone group: Tailored six-goal-oriented program designed by physiotherapist and surgeon focused on progressively increased functional stability training and activity modification where some sports, such as soccer, floorball, and wrestling, were advised against. Guided return	TAS score: ACLR median 4 (range 3-7), Rehab alone median 4 (range 2-7).<br><br>IPAQ: ACLR median 1563 (range 480–7572), Rehab alone median 1217 (range 212–7398), no difference between groups.	Mean 23 years (SD 2, range 17-28)

				Knee brace and crutches for 14 weeks.			to activity and work. The program aimed to achieve LSI >90% for strength and functional tests. Rehabilitation regarded as complete when the final step exercise could be performed without instability or other symptoms. The median time to reach this level was 22 weeks (range 12–60).		
Tsoukas et al 2016[16]	Prospective RCT	n=32<br><br>0% female	Randomisation to ACLR or rehab alone.<br><br>Time from injury to start of treatment: median 6 weeks (range 4–8) for both groups.	n=17<br><br>Median age 31 (range 20-36) years	n=15<br><br>Median age 33 (range 25-39) years	No reported difference in RTS advice.	Both groups: Initially knee extension brace, partial weight bearing and ROM exercises for 6 weeks. Next 6 weeks stationary bike, proprioception exercises, short arc quadriceps sets and hamstring curls. Allowed Jogging, swimming in straight line and bicycling at 3 months. Allowed pivot sports (e.g., ski, tennis and squash) at 6 months. Allowed contact sports at 8-9 months.	TAS score: ACLR median 7 (range 5-7), Rehab alone median 5 (range 3-7)	Median 10.1 years (range 10-11)

ACLR = Anterior Cruciate Ligament Reconstruction; D-ACLR = Delayed Anterior Cruciate Ligament Reconstruction; IRQ = interquartile range; LSI = Limb Symmetry Index; RCT = Randomised controlled trial; ROM = Range of movement; SD = Standard deviation; TAS = Tegner Activity Scale; IPAQ = International Physical Activity Questionnaire
