# Supplementary material for: No Difference in Return-to-Sport Rate or Activity Level in People with Anterior Cruciate Ligament (ACL) Injury Managed with ACL Reconstruction or Rehabilitation Alone: A Systematic Review and Meta-Analysis
Source: Sports Med. 2025 Jul 2;55(9):2191–205. doi: 10.1007/s40279-025-02268-5 (PMC12476414; doi:10.1007/s40279-025-02268-5)
Supplement: Supplementary file 3 — Supplementary file3 (PDF 48 KB) [file 40279_2025_2268_MOESM3_ESM.pdf]

Supplementary Appendix 3

Table 1. Return to pre-injury sport (%) within early ACLR, delayed ACLR, and rehabilitation-only subgroups

		Group size, return to pre-injury sport (%)		
		Early ACLR	Delayed ACLR	Rehab Only
Frobell et al., 2010	2 years	n=60, 44%	n=23, 30%	n=36, 39%
Frobell et al., 2013	5 years	n=59, 23%	n=30, 20%	n=29, 21%
Fithian et al., 2005	7 years	n=63, 52%	n=33, 37%	n=113, 52%
Pedersen et al 2021	5 years	n=135, 47%	n=23, 26%	n=64, 47%

Table 2. Tegner Activity Scale scores (mean(SD)) within early ACLR, delayed ACLR, and rehabilitation-only subgroups

		Group size, Tegner Activity Scale score mean (SD)		
		Early ACLR	Delayed ACLR	Rehab Only
Frobell et al., 2010	2 years	n=60, 5.8 (3.8)	n=23, 5.4 (2.4)	n=36, 5.7 (3.1)
Frobell et al., 2013	5 years	n=59, 4.4 (3.8)	n=30, 4.4 (3.9)	n=29, 4.2 (3.5)
