# Supplementary material for: No Difference in Return-to-Sport Rate or Activity Level in People with Anterior Cruciate Ligament (ACL) Injury Managed with ACL Reconstruction or Rehabilitation Alone: A Systematic Review and Meta-Analysis
Source: Sports Med. 2025 Jul 2;55(9):2191–205. doi: 10.1007/s40279-025-02268-5 (PMC12476414; doi:10.1007/s40279-025-02268-5)
Supplement: Supplementary file 4 — Supplementary file4 (PDF 46 KB) [file 40279_2025_2268_MOESM4_ESM.pdf]

Supplementary Appendix 4.

Risk of bias assessment results for Randomised Controlled Trials using ROB2.

Study	Intention to treat or per protocol	Randomisation process	Effect of assignment to intervention	Missing outcome data	Measurement of outcome**	Selection of reported result	Other concerns	Overall
Frobell 2010	Low<br>Intention to treat as not aware of anyone who refused ACLR surgery or anyone who had delayed ACLR if they didn't meet criteria	Low	Low	Low	Low	Low	No	Low
Frobell 2013	a/a	a/a	a/a	a/a	a/a	a/a		a/a
Tsoukas 2016	Low	Low	Low	Low	Low	Low	Serious.<br>Did not have ethics approval.<br>No delayed surgery or revisions in either group.<br>No details of how participants were recruited or number of refusals.<br>No flow chart.	Serious / unpredictable

** Outcomes were patient reported and assessor therefore not blinded to the intervention, however, our approach in this review is pragmatic and since it is impossible to blind participants in a surgery versus no surgery study, and in real life people will also always know whether they got surgery or not, studies were not downgraded due to assessor unblinding.
