# Supplementary material for: No Difference in Return-to-Sport Rate or Activity Level in People with Anterior Cruciate Ligament (ACL) Injury Managed with ACL Reconstruction or Rehabilitation Alone: A Systematic Review and Meta-Analysis
Source: Sports Med. 2025 Jul 2;55(9):2191–205. doi: 10.1007/s40279-025-02268-5 (PMC12476414; doi:10.1007/s40279-025-02268-5)
Supplement: Supplementary file 5 — Supplementary file5 (PDF 67 KB) [file 40279_2025_2268_MOESM5_ESM.pdf]

Supplementary Appendix 5.

Risk of bias assessment results for Non-Randomised Controlled Trials using ROBINS.

ROBINS-1 assessment of non-randomised trials:

**Study**	**Bias due to confounding** (e.g., Caused by unbalanced allocation to group, and/or unbalanced advice on RTS or activity)	**Bias in selection of participants into the study**	**Bias in classification of interventions**	**Bias due to deviations from intended interventions (effect of assignment to group)**	**Bias due to deviations from intended interventions (effect of adhering to allocated group)**	**Bias due to missing data**	**Bias in measurement of outcomes ****	**Bias in selection of the reported result**	**Overall risk of bias judgement / direction of bias**
Fink et al., 2001	Moderate / unknown	Low	Low	Low	Unclear	Moderate	Low	Low	Serious / unpredictable effect
	Surgery generally recommended for those under 40 years				No adherence data	37% lost to follow-up			
Fithian et al., 2005	Critical / probably favours surgery	Low	Low	Low	Unclear	Moderate	Low	Low	Critical / unpredictable effect
	Participants self-selected into group based on recommendation (based on 'risk' category) and their own preference				No adherence data	27% failed to attend follow-up			
Grindem et al., 2012	Critical / favours surgery	Low	Low	Low	Unclear	Low	Low	Low	Critical / favours surgery
	Group allocation determined by surgeon based on a				No adherence data				

	wish to return to level I sports, dynamic instability, young age and patient's preference. All participants level I sports pre-injury, but rehab-only group told not to return to level I sports								
Grindem et al., 2014	Critical / probably favours surgery	Low	Low	Low	Unclear	Low	Low	Low	Critical / favours surgery
	Patients chose surgical or non-surgical treatment. Adjusted analysis accounted for baseline confounders but not for post randomisation confounder of different RTS advice				No adherence data				
Keays et al., 2022	Critical / probably favours surgery	Low	Low	Low	Unclear	Low	Low	Low	Critical / favours surgery
	Rehab-only group elected not to have surgery due to work demands, lower physical demand, older age, surgeon's advice or because				No adherence data				

	they were functionally stable								
Kessler et al., 2008	Moderate / unpredictable effect	Low	Low	Low	Unclear	Low	Low	Low	Moderate / unpredictable effect
	Patients selected treatment after discussion with surgeon (not based on activity/sports participation, but no information on what factors were involved)				No adherence data				
Kovalak et al., 2018	Moderate / unpredictable effect	Low	Low	Low	Unclear	Low	Low	Low	Moderate / unpredictable effect
	Group allocation based on clinical and patient-related factors including fear of operative complications and occupation related issue	High level sports people excluded			No adherence data				
Meuffels et al., 2009<br><br>Van Yperen et al., 2018	Critical / probably favours surgery	Low	Low	Low	Unclear	Low	Low	Low	Critical / favours surgery
	Groups matched for pre-injury activity - ACLR patients were channelled into surgery because of instability at 3	Included only high-level sports people			No adherence data				

	months post injury; patients were offered either surgery OR non-pivoting-activity lifestyle								
Mihelic et al., 2010	Critical / probably favours surgery	Low	Low	Low	Unclear	Low	Low	Low	Critical / favours surgery
	Patients not taking up surgery told to modify sports and work activities	Only people under 40			No adherence data				
Moksnes et al., 2008/2009	Serious / unpredictable effect	Moderate / unpredictable	Low	Low	Unclear	Moderate	Low	Low	Serious / unpredictable
	Surgery was decided by surgeon based on activity level, type of activities, the number of giving-way episodes, age, the subject's own preferences	Inclusion in the study was at discretion of one surgeon involved in care			No adherence data	82% followed up; participants missing from the surgery group only.			
Pedersen et al, 2021	Serious / favours surgery	Low	Low	Low	Unclear	Moderate	Low	Low	Serious / favours surgery
	Group allocation based on patient choice including activity level and age	Included only high-level sports people			No adherence data	20% lost and almost no one lost from rehab only group (i.e., unbalanced missingness)			

Streich et al, 2011	Critical / favours surgery	Low	Low	Low	Unclear	Low	Low	Low	Critical / favours surgery
	Treatment based on clinical and patient-related factors - Rehab-only patients were instructed to avoid high demand pivot shift activities such as downhill skiing, soccer and comparable sports				No adherence data				
Tengman et al., 2014	Critical / favours surgery	Low	Low	Low	Unclear	Low	Low	Low	Critical / probably favours surgery
	Rehab-only group told to avoid certain sports but given very good rehab program - ACLR group rehab focused on return to sport				No adherence data				

** Outcomes were patient reported and assessor therefore not blinded to the intervention, however, our approach in this review is pragmatic and since it is impossible to blind participants in a surgery versus no surgery study, and in real life people will also always know whether they got surgery or not, studies were not downgraded due to assessor unblinding.

RTS = return to sport
