# Supplementary material for: No Difference in Return-to-Sport Rate or Activity Level in People with Anterior Cruciate Ligament (ACL) Injury Managed with ACL Reconstruction or Rehabilitation Alone: A Systematic Review and Meta-Analysis
Source: Sports Med. 2025 Jul 2;55(9):2191–205. doi: 10.1007/s40279-025-02268-5 (PMC12476414; doi:10.1007/s40279-025-02268-5)
Supplement: Supplementary file 6 — Supplementary file6 (PDF 473 KB) [file 40279_2025_2268_MOESM6_ESM.pdf]

Supplementary Appendix 6.

Study or Subgroup	ACLR Events	ACLR Total	Rehab only Events	Rehab only Total	Weight	Odds ratio IV, Random, 95% CI	Risk of bias
**1-4 years**							
Frobell et al. [9]	27	60	14	36	50.2%	1.29 [0.55 , 2.98]	+
Moksnes et al. [34]	35	50	36	52	49.8%	1.04 [0.45 , 2.41]	−
**Subtotal (Wald[a])**		**110**		**88**	**100.0%**	**1.16 [0.64 , 2.10]**	
Total events:	62		50				
Test for overall effect: Z = 0.47 (P = 0.64)							
Heterogeneity: Tau² (DL[b]) = 0.00; Chi² = 0.12, df = 1 (P = 0.72); I² = 0%							
**5-10 years**							
Fithian et al. [37]	33	63	58	113	28.5%	1.04 [0.56 , 1.93]	−
Frobell et al. [10]	13	59	6	29	21.9%	1.08 [0.36 , 3.22]	+
Keays et al. [27]	27	51	4	42	20.8%	10.69 [3.32 , 34.36]	−
Pedersen et al. [38]	64	135	30	64	28.8%	1.02 [0.56 , 1.85]	−
**Subtotal (Wald[a])**		**308**		**248**	**100.0%**	**1.70 [0.71 , 4.04]**	
Total events:	137		98				
Test for overall effect: Z = 1.19 (P = 0.23)							
Heterogeneity: Tau² (DL[b]) = 0.59; Chi² = 13.72, df = 3 (P = 0.003); I² = 78%							
**> 10 years**							
**Subtotal**		**0**		**0**		**Not estimable**	
Total events:	0		0				
Test for overall effect: Not applicable							
Heterogeneity: Not applicable							
Test for subgroup differences: Chi² = 0.51, df = 1 (P = 0.47), I² = 0%							

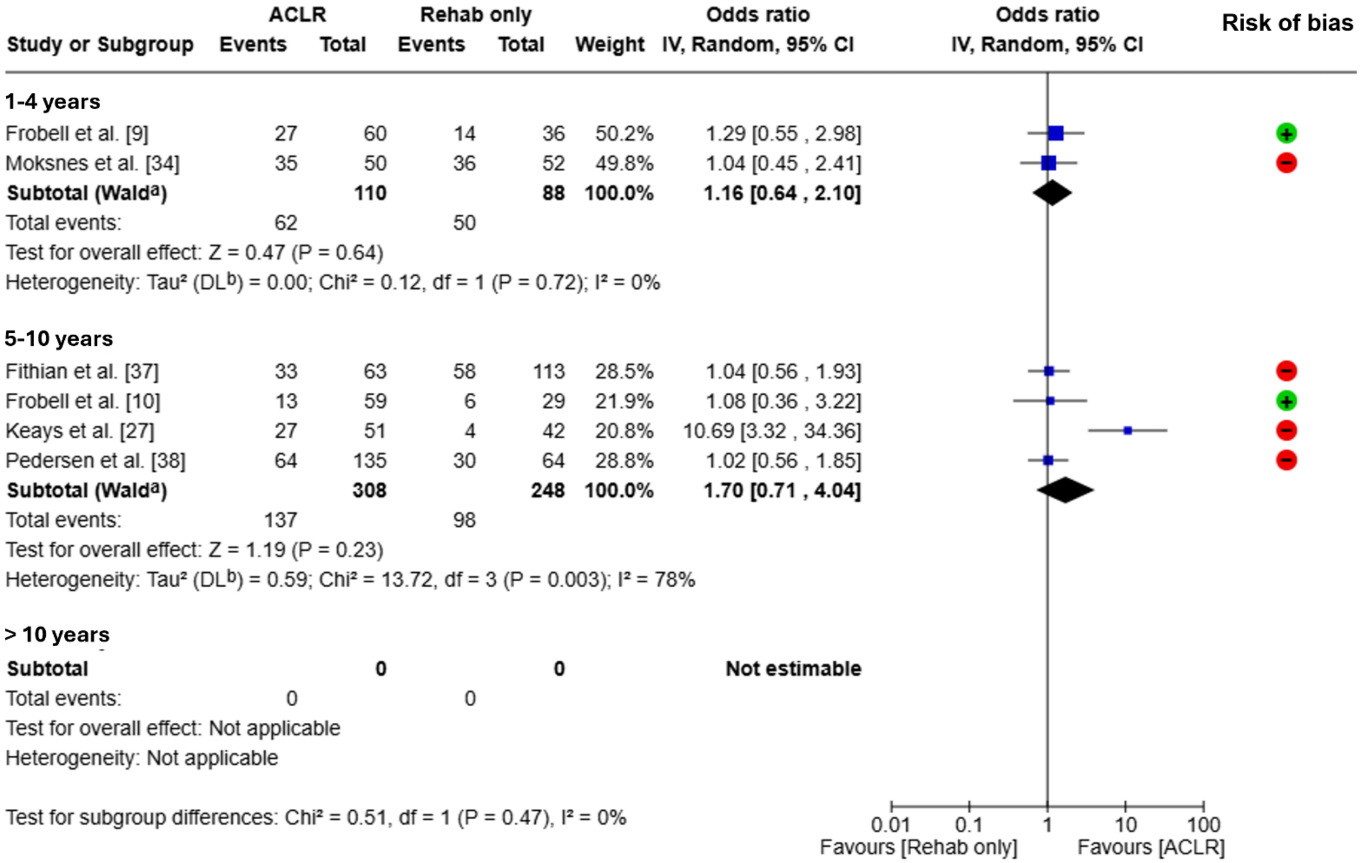

Footnotes

[a]CI calculated by Wald-type method.

[b]Tau² calculated by DerSimonian and Laird method.

Figure. Subgroup meta-analyses evaluating odds of returning to sport based on follow-up length

⊕ = overall low risk of bias; ⊖ = overall high risk of bias; ACLR = anterior cruciate ligament reconstruction; IV = inverse variance; CI = confidence interval
