# Supplementary material for: No Difference in Return-to-Sport Rate or Activity Level in People with Anterior Cruciate Ligament (ACL) Injury Managed with ACL Reconstruction or Rehabilitation Alone: A Systematic Review and Meta-Analysis
Source: Sports Med. 2025 Jul 2;55(9):2191–205. doi: 10.1007/s40279-025-02268-5 (PMC12476414; doi:10.1007/s40279-025-02268-5)
Supplement: Supplementary file 7 — Supplementary file7 (PDF 332 KB) [file 40279_2025_2268_MOESM7_ESM.pdf]

Supplementary Appendix 7.

Study or Subgroup	ACLR Mean [TAS score]	ACLR SD [TAS score]	ACLR Total	Rehab only Mean [TAS score]	Rehab only SD [TAS score]	Rehab only Total	Weight	Mean difference IV, Random, 95% CI [TAS score]
**1-4 years**								
Frobell et al. [9]	5.7935	3.7979	60	5.7108	3.0883	36	7.5%	0.08 [-1.31 , 1.48]
**Subtotal**			**60**			**36**	**7.5%**	**0.08 [-1.31 , 1.48]**
Test for overall effect: Z = 0.12 (P = 0.91)								
Heterogeneity: Not applicable								
**5-10 years**								
Frobell et al. [10]	4.3533	3.7995	59	4.1784	3.5088	29	6.3%	0.17 [-1.43 , 1.78]
Keays et al. [27]	6.8824	2.2418	51	5.5714	2.0499	42	11.4%	1.31 [0.44 , 2.18]
**Subtotal (Wald[a])**			**110**			**71**	**17.7%**	**0.95 [-0.09 , 1.99]**
Test for overall effect: Z = 1.80 (P = 0.07)								
Heterogeneity: Tau² (DL[b]) = 0.21; Chi² = 1.49, df = 1 (P = 0.22); I² = 33%								
**> 10 years**								
Kessler et al. [26]	5.3	2.2315	60	4.9	2.0431	49	12.0%	0.40 [-0.40 , 1.20]
Meuffels et al. [36]	6	1.5273	25	5	2.0364	25	10.3%	1.00 [0.00 , 2.00]
Mihelic et al. [41]	5.1126	1.6833	33	4	1.0978	18	12.3%	1.11 [0.35 , 1.88]
Streich et al. [42]	4.7	1.8	40	5.1	1.9	40	11.9%	-0.40 [-1.21 , 0.41]
Tengman et al. [43]	4.2251	0.9619	33	4.1053	1.1763	37	14.7%	0.12 [-0.38 , 0.62]
Tsoukas et al. [31]	6.6767	0.5567	17	5	1.1498	15	13.5%	1.68 [1.04 , 2.32]
**Subtotal (Wald[a])**			**208**			**184**	**74.8%**	**0.65 [0.02 , 1.28]**
Test for overall effect: Z = 2.01 (P = 0.04)								
Heterogeneity: Tau² (DL[b]) = 0.48; Chi² = 22.83, df = 5 (P = 0.0004); I² = 78%								
**Total (Wald[a])**			**378**			**291**	**100.0%**	**0.65 [0.15 , 1.16]**
Test for overall effect: Z = 2.52 (P = 0.01)								
Test for subgroup differences: Chi² = 0.96, df = 2 (P = 0.62), I² = 0%								
Heterogeneity: Tau² (DL[b]) = 0.39; Chi² = 26.06, df = 8 (P = 0.001); I² = 69%								

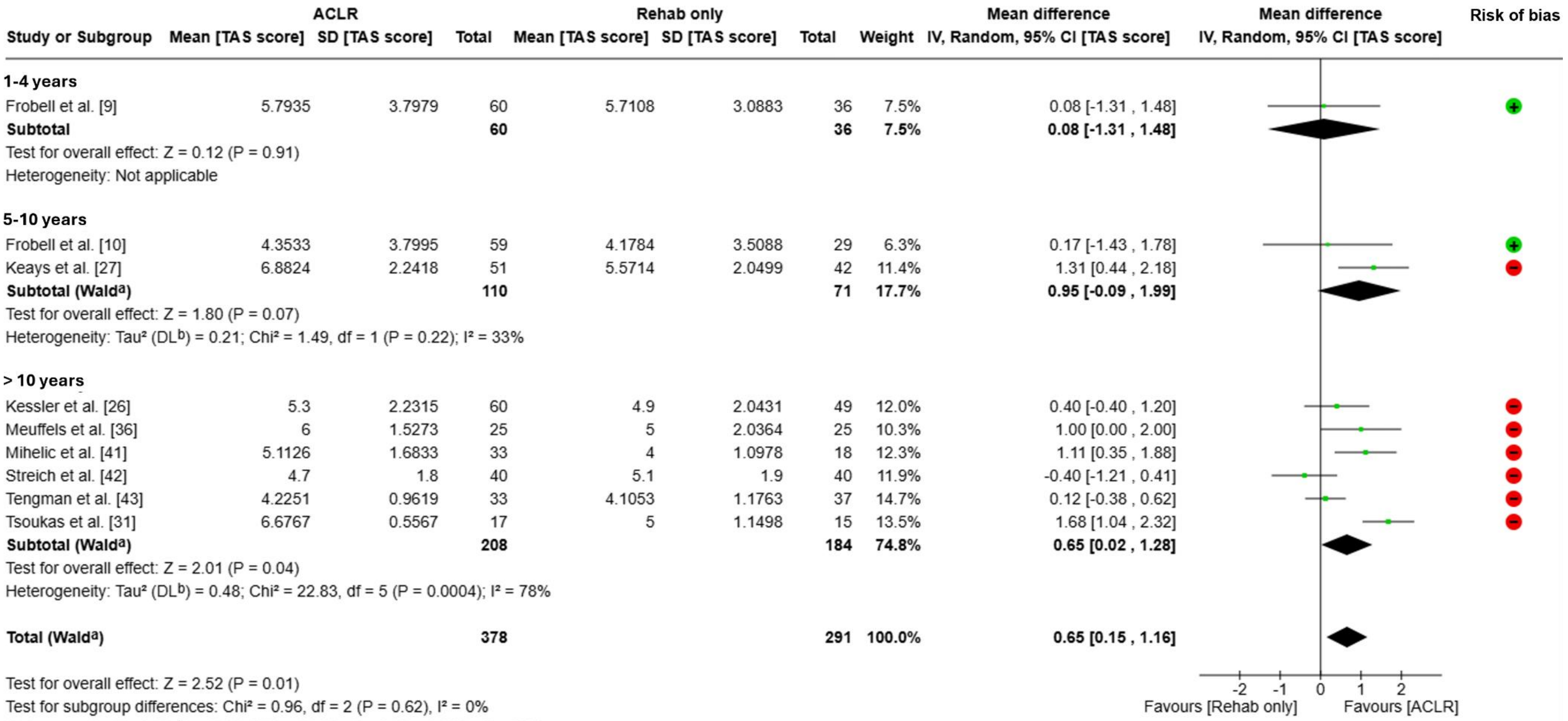

Footnotes

[a]CI calculated by Wald-type method.

[b]Tau² calculated by DerSimonian and Laird method.

Figure. Subgroup meta-analyses evaluating activity levels (measured by Tegner Activity Scale), based on follow-up length

⊕ = overall low risk of bias; ⊖ = overall high risk of bias; ACLR = anterior cruciate ligament reconstruction; TAS = Tegner Activity Scale IV = inverse variance; CI = confidence interval; SD = standard deviation
